# Characterization of *salA*, *syrF*, and *syrG* Genes and Attendant Regulatory Networks Involved in Plant Pathogenesis by *Pseudomonas syringae* pv. *syringae* B728a

**DOI:** 10.1371/journal.pone.0150234

**Published:** 2016-03-08

**Authors:** Vanessa L. Vaughn, Dennis C. Gross

**Affiliations:** Department of Plant Pathology and Microbiology, Texas A&M University, College Station, Texas, United States of America; Virginia Tech, UNITED STATES

## Abstract

*Pseudomonas syringae* pv. *syringae* B728a, causal agent of brown spot on bean, is an economically important plant pathogen that utilizes extracellular signaling to initiate a lifestyle change from an epiphyte to a pathogen. LuxR regulatory proteins play an important role in the transcriptional regulation of a variety of biological processes involving two-component signaling, quorum sensing, and secondary metabolism. Analysis of the B728a genome identified 24 LuxR-like proteins, three of which are encoded by *salA*, *syrF*, and *syrG* located adjacent to the syringomycin gene cluster. The LuxR-like proteins encoded by these three genes exhibit a domain architecture that places them in a subfamily of LuxR-like proteins associated with regulation of secondary metabolism in B728a. Deletion mutants of *salA*, *syrF*, and *syrG* failed to produce syringomycin and displayed reduction of virulence on bean. The transcriptional start sites of *salA*, *syrG*, and *syrF* were located 63, 235, and 498 bp upstream of the start codons, respectively, using primer extension analysis. The predicted -10/-35 promoter regions of *syrF* and *syrG* were confirmed using site-directed mutagenesis and GFP reporters that showed conserved promoter sequences around the -35 promoter region. Overexpression analysis and GFP reporters identified SyrG as an upstream transcriptional activator of *syrF*, where both SyrG and SyrF activate promoters of syringomycin biosynthesis genes. This study shows that *syrG* and *syrF* encode important transcriptional regulators of syringomycin biosynthesis genes.

## Introduction

*P*. *syringae* pv. *syringae* (*Pss*) B728a [1, 2] is an aggressive plant pathogen of bean that causes brown spot. *Pss* B728a is highly adapted to its host where it has the ability to function as an epiphyte on leaf surfaces before invading apoplastic tissues as a plant pathogen. The bacterium’s pronounced epiphytic phase produces large bacterial populations residing on the surfaces of bean leaves, where it persists until it utilizes extracellular signaling to initiate a lifestyle change from an epiphyte to a pathogen [3]. Epiphytic populations provide a source of inoculum that is used to colonize the apoplast under appropriate conditions and multiply by using nutrients available in living host cells [4]. During apoplastic colonization, *Pss* B728a extensively expresses genes associated with pathogenicity and virulence that include type III secretion systems, exopolysaccharides, siderophores, an ice nucleation protein, cell wall-degrading enzymes, and phytotoxins [5]. The molecular basis for the switch from an epiphyte to pathogen is complex requiring the intricate interaction and regulation of multiple virulence factors, which makes *Pss* B728a an important model in the study of molecular plant pathogenesis [1, 2]. The lipopetide phytotoxins, syringomycin and syringopeptin, are considered important virulence factors that contribute to the disease development of brown spot on bean. The phytotoxins function by inserting into the cell membrane of the host to form small pores that result in electrolyte leakage and cell death [6–8]. Genes responsible for the biosynthesis of syringomycin and syringopeptin are encoded on two adjacent gene clusters referred to as the *syr-syp* gene cluster [6, 7, 9]. The *syr-syp* gene cluster found in *Pss* B728a displays similar size and gene organization as *Pss* B301D. Adjacent to the *syr-syp* gene cluster are three transcriptional regulatory genes, *salA*, *syrF*, and *syrG* that were identified as encoding LuxR-like proteins [10]. These LuxR-like proteins have been implicated in virulence and syringomycin regulation [10].

**Fig 1:**
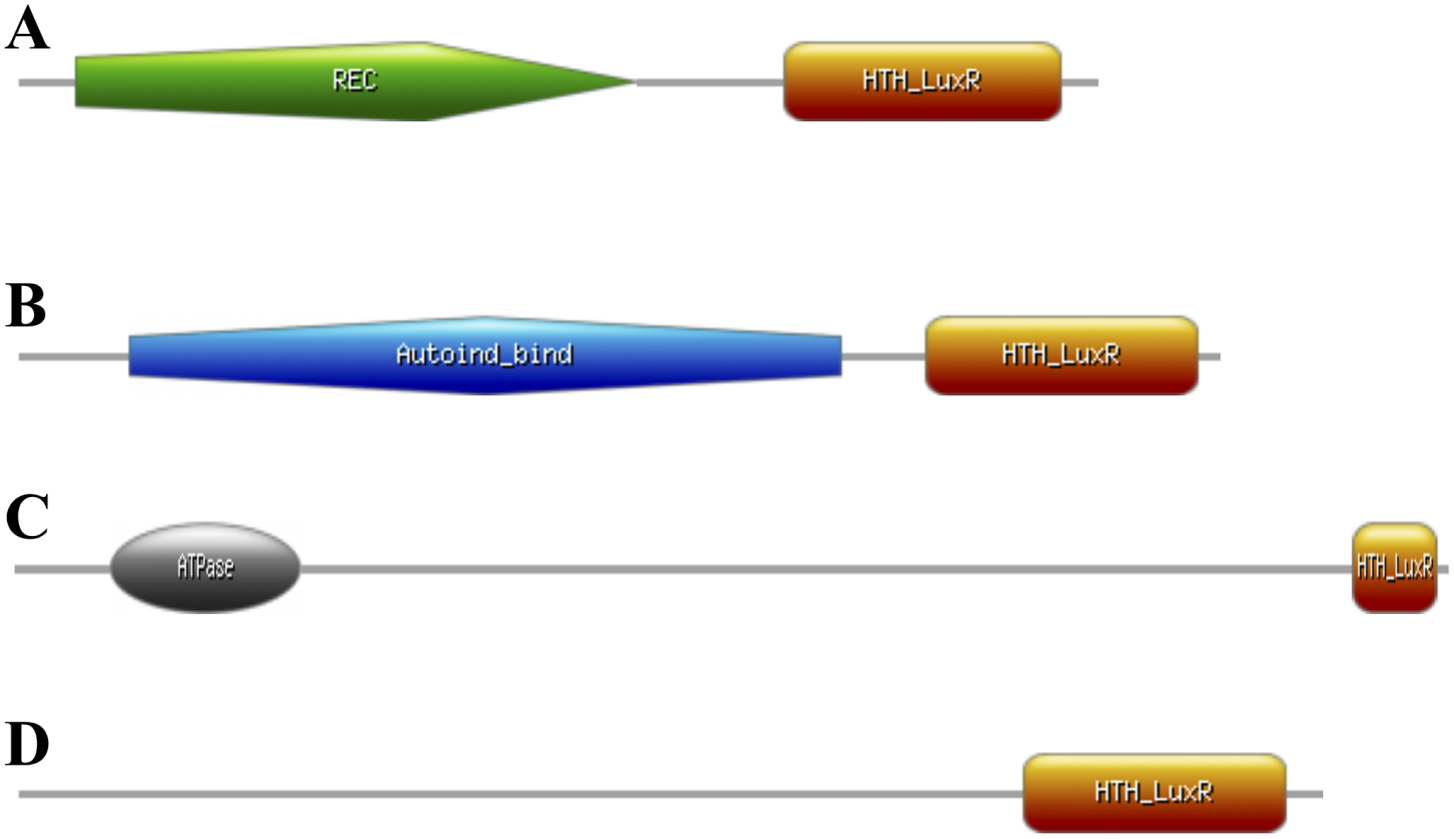
Domain organization of LuxR proteins that are classified into four sub-families based on domain architecture and mechanism of regulatory activation. **A.** GacA is a LuxR-like protein in *Pss* B728a part of a global signal transduction system characterized as having an N-terminal receiver domain activated by phosphorylated and an C-terminal HTH DNA-binding domain that is characteristic of the first sub-family of LuxR-like proteins. **B.** AhlR is part of quorum sensing system in *Pss* B728a with AhlI. It has an N-terminal auto-inducer binding domain where hexanoyl-homoserine lactone binds to activate transcription of *ahlI* and has a C-terminal HTH DNA-binding domain. This domain organization is typical of the second sub-family of LuxRs associated with quorum sensing. **C.** *Psyr_0993*, which has not been characterized in *Pss* B728a, shares homology to *malT* in *E*. *coli*. These genes encode a subfamily of LuxR-like proteins have an N-terminal AAA ATPase domain that requires ATP for transcriptional activation and has a C-terminal HTH DNA binding domain. **D.** SyrG, which has been implicated in virulence and syringomycin production in *Pss* B728a lacks any defined N-terminal regulatory domain and has a C-terminal HTH DNA binding domain. This domain organization is typically seen in the fourth subfamily of LuxR-like proteins, which have not been fully defined functionally. LuxR-like proteins characterized in this family of LuxRs have been associated with secondary metabolism in *Pss* B728a.

LuxR proteins are a family of prokaryotic transcriptional regulators that are defined by having a helix-turn-helix (HTH) DNA binding motif on the C-terminus region of the protein and an N-terminus response regulatory domain [11–13]. The LuxR superfamily can be grouped into four subfamilies based on domain architecture and the mechanism of regulatory activation, illustrated in Fig 1 [12]. The first subfamily consists of regulators that are part of a two-component sensory transduction system that are activated by the phosphorylation of an aspartate residue on the N-terminal region of the protein, typically by a transmembrane kinase. An example of this subfamily of LuxR is NarL [14, 15], which activates the nitrate reductase operon in *E*. *coli*. NarL is comprised of two domains, an N-terminal receiver domain that is controlled by phosphorylation and a C-terminal effector domain that elicits a physiological response. Phosphorylation occurs at the N-terminal domain to form dimers that recognize heptamer sequences in the promoter regions of gene targets [12, 14, 15]. Regulators activated by *N*-acyl homoserine lactone comprise the second subfamily of LuxR proteins, which includes LuxR [12, 16], TraR [12, 17], CarR [12, 18], ExpR [19], LasR [12, 20], PhzR [21], and RhlR [22]. LuxR is involved in the activation of bioluminescence related genes and is essential for quorum sensing in *Vibrio fischeri* [23]. These regulators have a C-terminal HTH DNA binding domain and an N-terminal autoinducer-binding domain that interacts with acyl-homoserine lactone, which is a signaling molecule involved in quorum sensing. The third subfamily of regulators is referred to as large ATP-binding regulators of the LuxR family (LAL) [24–26]. Experimentally characterized LALs include GdmRI [25], GdmRII [25], MalT [24], and PikD [26]. The most studied LAL is MalT, which is the transcriptional activator of the maltose regulon in *E*. *coli* but requires two co-factors for activation [24]. This subfamily of LuxR proteins is significantly different because they are relatively large in size (800 to 1,200 amino acids), contain an N-terminal ATP-binding motif, and contain a C-terminal HTH DNA binding domain [24–26]. These LuxR proteins require the binding of ATP to the N-terminal region for activation. The fourth subfamily of regulators represents the simplest form of the LuxR superfamily because they harbor the typical C-terminal HTH DNA binding domain but lack an N-terminal regulatory domain. GerE was one of the first transcriptional regulators placed into this group of LuxRs [27]. This regulator was involved in the transcriptional regulation of genes associated with spore formation and maturation in *Bacillus subtilis* [27].

Bioinformatic investigation of the 6.09-Mb genome of *Pss* B728a revealed 24 genes encoding LuxR-like proteins dispersed throughout the genome, homologs of these LuxR-like proteins were also found in the genome of *Pss* B301D. The genes *gacA* (*Psyr_2897*), *Psyr_1294*, *Psyr_1384*, *Psyr_1940*, *Psyr_2114*, *Psyr_3299*, *Psyr_3890*, *Psyr_4376*, *Psyr_4618*, and *Psyr_5088* encode proteins that were identified as belonging to a subfamily of LuxRs that are typically part of a two-component sensory transduction system. This subfamily is one of the largest groups of LuxR proteins found in *Pss* B728a. The LuxR-like proteins encoded on *ahlR* (*Psyr_1622*), *Psyr_1858*, and *Psyr_4216* are classified as belonging to the second subfamily of LuxR proteins, associated with quorum sensing. The third and smallest group of LuxRs found in *Pss* B728a belongs to the third subfamily of LuxR proteins, referred to as LAL, which includes only one LuxR protein encoded on *Psyr_0993*. *Psyr_0993* has not been functionally defined in *Pss* B728a but does encode a protein that exhibits domain architecture typical of this subfamily of LuxR proteins. The proteins that are encoded by *salA* (*Psyr_2601*), *sylA* (*Psyr_1702*), *syrG* (*Psyr_2602*), *syrF* (*Psyr_2607*), *syrR* (*Psyr_2575)*, *Psyr_2045*, *Psyr_2578*, *Psyr_3767*, *Psyr_4266*, and *Psyr_4278* exhibit domain architecture that is typical of the fourth subfamily of LuxR proteins, which is the second largest group of LuxRs found in the *Pss* B728a genome. These transcriptional regulators seemingly play a key role in the regulation of genes associated with secondary metabolism, pathogenicity, and virulence of *Pss* B728a.

The LuxR-like proteins SalA, SyrF, and SyrG are part of the fourth subfamily of LuxR proteins, which are not completely defined [10, 12]. SalA is part of a complex regulatory network that is involved in the biosynthesis, secretion, and regulation of syringomycin, syringopeptin, and syringolin [28]. All genes identified to be part of the SalA regulon are absent from the genome of *Pst* DC3000. This transcriptional regulator is under the control of the GacS/GacA global signal transduction system, which controls expression of genes essential for plant pathogenesis [29]. Also, it has been demonstrated that SalA is required for the functional activation of both *syrG* and *syrF* [10]. Both *salA* and *syrF* genes are necessary for the biosynthesis of syringomycin and syringopeptin, which led to the conclusion that the regulatory networks involving syringomycin and syringopeptin overlap, but are not identical [10]. Meanwhile, SalA mediates the regulation of syringomycin and syringopeptin through the regulation of *syrF* [12]. Protein sequence analysis also revealed that both SyrF and SyrG have somewhat similar protein sequences with 49% identity [10]. The sequence similarity is significant given that SalA only has 27% and 26% identity to the protein sequence of SyrF and SyrG. The similarity of SyrF and SyrG may indicate similar regulatory gene targets. Previous research established that both *salA* and *syrF* genes are required for syringomycin production, where *syrG* gene expression is highly induced in the apoplast and is associated with virulence [5, 10]. It is surmised that SyrG plays a critical role in the regulation of genes associated with pathogenesis given that mutants of *syrG* displayed a significant reduction in virulence [10]. It appears that the regulatory role of SyrG in virulence is complex and may involve molecular mechanisms that may reside outside the *syr-syp* gene cluster.

Despite previous evidence that *salA*, *syrF*, and *syrG* genes have an effect on virulence and syringomycin production [10], the regulatory role of SyrG in regards to syringomycin production, and the production of other secondary metabolites, remains unknown. In addition, the previous study performed by Lu et al. [10] utilized site directed insertional mutants of *salA*, *syrF*, and *syrG* in *Pss* B301D to determine their effect on virulence and syringomycin production. It is hypothesized that these insertional mutants still produce truncated proteins with reduced functional activity displaying low levels of toxin production instead of eliminating it completely. It was important to test this hypothesis by determining the effect *salA*, *syrF*, and *syrG* in *Pss* B728a has on virulence and syringomycin production with clean deletions mutants. Also, it is hypothesized that the LuxR-like protein SyrG is involved in the regulation of genes essential for the pathogenic lifestyle of *Pss* B728a while under transcriptional control of SalA. This hypothesis was tested utilizing phenotypic characterization and quantitative real-time PCR analysis in an effort to identify new components of the SyrG regulon. It was demonstrated in this study that the *syrG* gene has a stronger influence on virulence and phytotoxin production than previously reported [10]. It was illustrated that SyrG is required for virulence but is not required for the replication of *Pss* B728a *in planta*. The LuxR-like protein, SyrG, is not involved in the transcriptional regulation of known virulence genes associated with the biosynthesis of achromobactin, alginate, levansucrase, pyoverdine, syringolin, and syringafactin. However, both SyrG and SyrF are required for syringomycin production with SyrG being an important transcriptional regulator of genes associated with the biosynthesis of syringomycin in *Pss* B728a. It has been established that SalA controls the expression of both *syrG* and *syrF* [10], but it is unknown how SyrG affects the expression of *syrF* and vice versa. It is important to further define these transcriptional regulators in order to fully understand the complex nature of SalA, SyrG, and SyrF regulons in regards to virulence and the plant-pathogen interaction.

## Materials and Methods

### Bacterial strains, plasmids and media

Bacterial strains and plasmids used in this study are listed in Table 1. One Shot^®^ TOP10 chemically competent *E*. *coli* cells were used for cloning reactions following manufacturer’s protocols (Invitrogen, Carlsbad, CA). *Pss* B728a and mutant strains were cultured from 20% glycerol stocks stored at -80°C onto nutrient broth-yeast extract (NBY) [30], or on King’s B (KB) [31] at 26°C. Bioassays for syringomycin were grown on Hrp minimal medium (HMM) agar [32, 33]. The following antibiotic concentractions (μg/ml) were added to media: rifampicin, 100; kanamycin, 75; tetracycline, 20; ampicillin, 100; gentamycin, 5; spectinomycin, 100.

**Table 1:** Strains and plasmids.

Designation	Relevant characteristics	Source
Bacterial Strains		
*Escherichia coli*		
One Shot^®^ TOP10	F- *mcr*A Δ(*mrr-hsd*RMS-*mcr*BC)φ80*lac*ZΔM15 Δ*lac*X74 *rec*A1 *ara*D139 Δ(*ara-leu*)7697 *gal*U *gal*K *rps*L (Str^R^) *end*A1 *nup*G	Invitrogen
*P*. *syringae* pv. *syringae*		
B728a	Wild-type, bean pathogen; Rif^r^	[1]
B728aΔ*salA*	*salA* mutant derivative of B728a, Rif^r^	[34, 35]
B728aΔ*syrF*	*syrF* mutant derivative of B728a, Rif^r^	This study
B728aΔ*syrG*	*syrG* mutant derivative of B728a, Rif^r^	This study
B728aΔ*syrF*Δ*syrG*	*syrF* and *syrG* mutant derivative of B728a, Rif^r^	This study
B728aΔ*gacS*	*gacS* mutant derivative of B728a, Rif^r^	[36]
Plasmids		
pE2602	pENTR/D-TOPO 6.80 kb region carrying *syrG*, Km^r^	This study
pE2607	pENTR/D-TOPO 6.62-kb region carrying *syrF*, Km^r^	This study
pKD13	Template plasmid containing FRT-flanked *nptII*	[37]
pLVCD	Gateway destination vector for mating with *P*. *syringae*; pBR322 derivative with *mob* genes from RSF1010; Tc^r^ Ap^r^ Cm^r^	[38]
pLV2602	pLVCD carrying *syrG*; Tc^r^ Ap^r^	This study
pLV2607	pLVCD carrying *syrF*; Tc^r^ Ap^r^	This study
pLV2602-FP	pLVCD carrying upstream and downstream regions of *syrG* fused to *nptII*; Tc^r^ Ap^r^ Km^r^	This study
pLV2607-FP	pLVCD carrying upstream and downstream regions of *syrF* fused to *nptII*; Tc^r^ Ap^r^ Km^r^	This study
pPROBE-KT’	Promoter-probe vector with pVS1/p15a replicon and *gfp* reporter, Km^r^	[39]
pPKT::*syrG*752	pPROBE-KT’ carrying *syrG* along with 752-bp upstream; Km^r^	This study
pPKT::*syrG*552	pPROBE-KT’ carrying *syrG* along with 552-bp upstream; Km^r^	This study
pPKT::*syrG*452	pPROBE-KT’ carrying *syrG* along with 452-bp upstream; Km^r^	This study
pPKT::*syrG*352	pPROBE-KT’ carrying *syrG* along with 352-bp upstream; Km^r^	This study
pPKT::*syrG*352-10	pPROBE-KT’ carrying *syrG* along with 352-bp upstream with the potential -10 region replaced with CTGCAG; Km^r^	This study
pPKT::*syrG*352-35	pPROBE-KT’ carrying *syrG* along with 352-bpupstream with the potential -35 region replaced with CTGCAG; Km^r^	This study
pPKT::*syrG*252	pPROBE-KT’ carrying *syrG* along with 252-bp upstream; Km^r^	This study
pPKT::*syrG*202	pPROBE-KT’ carrying *syrG* along with 202-bp upstream; Km^r^	This study
pPKT::*syrG*102	pPROBE-KT’ carrying *syrG* along with 102-bp upstream; Km^r^	This study
pPKT::*syrG*52	pPROBE-KT’ carrying syrG along with 52-bp upstream, Km^r^	This study
pPKT::*syrF*	pPROBE-KT’ carrying *syrF* along with 1.3-kb upstream; Km^r^	This study
pPKT::*syrF*1000	pPROBE-KT’ carrying *syrF* along with 1.0-kb upstream; Km^r^	This study
pPKT::*syrF*800	pPROBE-KT’ carrying *syrF* along with 800-bp upstream; Km^r^	This study
pPKT::*syrF*600	pPROBE-KT’ carrying *syrF* along with 600-bp upstream; Km^r^	This study
pPKT::*syrF*600-10	pPROBE-KT’ carrying *syrF* along with 600-bp upstream with the potential -10 region replaced with CTGCAG; Km^r^	This study
pPKT::*syrF*600-35	pPROBE-KT’ carrying *syrF* along with 600-bp upstream with the potential -35 region replaced with CTGCAG; Km^r^	This study
pPKT::*syrF*500	pPROBE-KT’ carrying *syrF* along with 500-bp upstream; Km^r^	This study
pPKT::*syrF*252	pPROBE-KT’ carrying *syrF* along with 252-bp upstream; Km^r^	This study
pPKT::*syrF*202	pPROBE-KT’ carrying *syrF* along with 202-bp upstream; Km^r^	This study
pPKT::*syrF*152	pPROBE-KT’ carrying *syrF* along with 152-bp upstream; Km	This study
pPKT::*syrF*102	pPROBE-KT’ carrying *syrF* along with 102-bp upstream; Km^r^	This study
pPKT::*syrF*52	pPROBE-KT’ carrying *syrF* along with 52-bp upstream; Km^r^	This study
pMEKm12	*E*. *coli* and *P*. *syringae* pv. *syringae* overexpression vector, Km^r^	[40]
pMK::*syrG*	pMEKm12 carrying the *syrG* gene in-frame fused to *malE*; Km^r^	This study
pMK::*syrG*583	pMEKm12 carrying 583-bp of the *syrG* N-terminal region fused to *malE*; Km^r^	This study
pMK::*syrF*	pMEKm12 carrying the *syrF* gene in-frame fused to *malE*; Km^r^	This study
pMK::*syrF*583	pMEKm12 carrying 583-bp of the *syrF* N-terminal region fused to *malE*; Km^r^	This study
pRK2073	Helper plasmid; Sp^r^ Trm^r^	[41]

### General DNA manipulations

For methodologies that involve the use of Gateway cloning technology ([42] and regulatory aspects of lambda site specific recombination), targeted genes were PCR amplified and cloned into the pENTR/D-TOPO vector following the manufacturer’s protocols (Invitrogen). Recombination between pENTR constructs and Gateway destination vectors was performed employing the use of LR clonase in accordance with the manufacturer’s protocol (Invitrogen). Plasmids were introduced into *E*. *coli* by chemical transformation or electroporation [43]. Plasmids were incorporated into *P*. *syringae* by tri-parental mating utilizing the helper plasmid pRK2073 [41]. Complementation of *Pss* B728a derivative mutants was achieved by the electroporation of the complement construct. Standard PCR procedures and cycling conditions were used [32, 36].

Restriction enzymes, and T4 DNA ligase were purchased from New England Biolabs (Beverly, MA). Phusion High-Fidelity DNA polymerase was purchased from Thermo Scientific Inc. (Waltham, MA). In-Fusion^®^ HD cloning kit was purchased from Clontech Laboratories (Mountain View, CA). The design and purchase of oligonucleotides was acquired using PrimerQuest and OligoAnalyzer applications of Integrated DNA technologies (Coralville, IA). The oligonucleotide sequences are listed in S1 Table. Plasmids were introduced into *E*. *coli* by chemical transformation or electroporation [43]. Plasmids were transferred to *P*. *syringae* by triparental mating using the helper plasmid pRK2073 [41]. Standard PCR procedures and cycling conditions were used.

### Bioinformatic analysis

Protein sequences were retrieved using the *Pseudomonas* Genome Database [44]. The Conserved Domain Database at NCBI (http://www.ncbi.nlm.nih.gov/Structure/cdd/) was used to identify conserved domains of protein sequences. Additionally, database searches were performed using a Basic Local Alignment Search Tool (BLAST) to identify homologous sequences SyrG and SyrF in pseudomonad genomes. A clustalW alignment of homologous protein sequences was generated using the CLC Genomics Workbench (V5.5, CLC Bio.) [32].

### Construction of markerless deletion mutants in *Pss* B728a

For targeted deletion mutants in *Pss* B728a, the gene of interest (GOI) along with 3 to 4-kb of flanking DNA was PCR amplified using Phusion^®^ high fidelity polymerase (ThermoScientific). The purified PCR product was cloned into a Gateway entry vector pENTR/D-TOPO (Invitrogen) and transformed chemically into *E*. *coli* One Shot^®^ TOP10 cells. LR clonase II (Invitrogen) was used to carry out recombination between the pENTR construct and the *Pseudomonas* suicide vector, pLVC-D [38].

Site-directed mutagenesis occurred by linearization of the pLVC-D plasmid (pLVC-D:flank-GOI-flank) using inverse PCR with primers that exclude the GOI, and purified using a Wizard^®^ SV Gel and PCR Clean-Up System (Promega, Madison, WI). A linear kanamycin cassette (*nptII*) flanked by the FLP recognition target sites, was amplified from pKD13 plasmid using primers with 15 bp extensions that were homologous to regions adjacent to the GOI [37]. The kanamycin cassette was cloned into the purified linearized pLVC-D construct using the In-Fusion^®^ HD cloning kit (Clontech) according to the manufacturer’s protocol and chemically transformed into *E*. *coli* One Shot^®^ TOP10 cells for confirmation of the construct. The resulting pLVC-D construct (pLVC-D:flank-*nptII*-flank) was moved into *Pss* B728a by triparental mating with the helper plasmid pRK2073 [41]. Colony PCR and qRT-PCR was used to confirm double recombination of the kanamycin cassette into *Pss* B728a, replacing the GOI. The kanamycin marker was later removed by the introduction of the pBH474 vector carrying the FLP recombinase gene. FLP recombination resulted in the loss of the *nptII* marker, giving a markerless deletion mutant in *Pss* B728a. The Suc^s^ pBH474 plasmid was cured from the B728a deletion mutant cells by culturing in NBY + 5% sucrose liquid medium.

### Construction of complementing and overexpressing plasmids

For the complementation of B728a derivative mutants a copy of the targeted gene and the predicted promoter region was PCR amplified from *Pss* B728a with a *Bam*HI and *Sac*I restriction enzyme sited on each end of the PCR product using primers listed in S1 Table. The PCR product was digested with *Bam*HI and *Sac*I. Additionally, the broad-host-range promoter-probe vector, pPROBE-KT’ was digested with *Bam*HI and *Sac*I [39]. Digested PCR products and vector were purified using Wizard^®^ SV Gel and PCR Clean-Up System (Promega). Purified digested products were quantified using micro-spectrophotometry (Nano-Drop Technologies, Inc.). Ligation of the vector and insert was performed using T4 DNA ligase (New England Biolabs) and chemically transformed into *E*. *coli* One Shot^®^ TOP10 cells (Invitrogen) for confirmation of construct. Both constructs, pPROBE-KT’:*syrF* and pPROBE-KT’:*syrG*, were introduced into B728a derivative mutants by electroporation.

The overexpression of SyrF, SyrG, and their respective truncated proteins missing portions of the C-terminal region were cloned into the expression vector, pMEKm12 [40]. A total of two overexpression constructs was generated for each gene of interest. For *syrF*, the targeted gene, and 583-bp of the N-terminal sequence was PCR amplified from *Pss* B728a with *Bam*HI and *Xba*I restriction enzyme sites flanking the PCR products. For *syrG*, the targeted gene, and 583-bp of the N-terminal sequence was PCR amplified from *Pss* B728a with *Bam*HI and *Xba*I restriction enzyme sites flanking the PCR products. The PCR products and the expression vector, pMEKm12, were digested with *Bam*HI and *Xba*I. Digested PCR products and vector were purified using Wizard^®^ SV Gel and PCR Clean-Up System (Promega). Purified digested products were quantified using micro-spectrophotometry (Nano-Drop Technologies, Inc.). Ligation of the vector and insert was performed using T4 DNA ligase (New England Biolabs) and chemically transformed into *E*. *coli* One Shot^®^ TOP10 cells (Invitrogen) for confirmation of construct. Overexpression constructs were introduced into *Pss* B728a by electroporation.

### Pathogenicity assays

The ability of derivative mutants (B728aΔ*salA*, B728aΔ*syrF*, B728aΔ*syrG*, and B728aΔ*syrF*Δ*syrG*) to cause disease and multiply *in planta* was evaluated by vacuum infiltration on 2-week old Blue Lake 274 (Burpee Seeds, Warminster, PA) bean plants (*Phaseolus vulgaris* L.) and 4-week old *Nicotiana benthamiana*. The method for vacuum infiltration was described previously [32, 36]. *Pss* B728a was used as a positive control and *Pss* B728aΔ*gacS* served as the negative control. Each strain was evaluated on at least three plants of each species, with triplicate biological replicates. The virulence of derivative mutants was evaluated by measurement of necrotic lesion surface area found on leaves 3 days post inoculation. Lesion surface areas were calculated using ImageJ software (version 1.49e; http://rsbweb.nih.gov/ij/). A total of three leaves were evaluated for each biological replicate.

To evaluate the ability of derivative mutants to replicate *in planta*, population analysis was performed for B728a, B728aΔ*salA*, B728aΔ*syrF*, B728aΔ*syrG*, B728aΔ*syrF*Δ*syrG*, and B728aΔ*gacS* on Day 0 and Day 3 after vacuum infiltration of bean plants. From each infiltrated plant, a trifoliate leaf was detached and infiltrated tissue was removed using the bottom of a sterile 2 mL microcentrifuge tube (Bio Plas Inc., San Franscisco, CA). A total of 10 leaf discs were removed per leaf and rinsed with sterile deionized water. The leaf discs were ground using a sterile mortar and pestle with Silwet Phosphate Magnesium Buffer (SPM) [32]. Serial dilutions were prepared with SPM buffer and plated on KB agar with appropriate antibiotics followed by incubation at 26°C for 48 h. Colonies were counted and calculated as CFU per squared cm.

### Syringomycin assays

The production of syringomycin by *Pss* B728a and derivative mutant strains were evaluated using a bioassay previously described [45] for syringomycin production on HMM agar. Bacterial strains were grown overnight in 2 ml NBY at 26°C with shaking at 180 rpm. Cells were washed and resuspended in sterile deionized water to OD_600_ = 0.3 (~2 x 10^8^ CFU/ml), and 5 μl aliquots of bacterial suspension were spotted on HMM. After an incubation period of 3 days at 26°C the plates were lightly sprayed with a cell suspension of *Geotrichum candidum* strain F-260 using a sterile chromatography sprayer. After 24 h, quantification of syringomycin production was determined by measuring the diameter of inhibition zones and compared to the parental strain of *Pss* B728a. This experiment was repeated in triplicate.

### RNA isolation

Pss B728a and derivative mutant strains were cultured overnight with shaking at 26°C in 5 ml of liquid NBY medium. Cells were harvested by centrifugation, washed and resuspended in sterile deionized water to a concentration of approximately 2 x 10^8^ CFU per ml. Cell suspensions (100 μl) were spread onto HMM agar and incubated at 26°C for 48 h. Total RNA was purified using an RNeasy Mini Kit along with the RNAprotect reagent following the manufacturer’s protocol (Qiagen Inc., Valencia, CA). For studies that evaluated the influence of the apoplast on gene expression, bacterial strains were introduced into bean leaves by vacuum infiltration. Approximately, 48 hours post-inoculation, a total of 40–80 leaves were harvested individually and endophytic bacteria were extracted using an acidic ethanol/phenol solution (9 mL buffer-saturated phenol (pH 6.6), 171 mL absolute ethanol, 420 mL sterile distilled water). The leaves were individualy removed from the plants and cut into squares (~3 x 3 mm^2^) while submerged in the ethanol/phenol, and the plant tissue and liquid were sonicated for 10 min. The solution was filtered through sterile cheese cloth and centrifuge to remove bacterial cells at 5,500 x g for 10 min. The cell and plant debris pellet was resuspended in approximately 5 mL of the supernatant and filtered through a Luer-Lock syringe packed with sterile cheesecloth and fitted with a Millipore Millex 25 mm Durapore^®^ PVDF 5 μm filter unit. Cells were pelleted, flash frozen and stored up to 2 weeks at -20C prior to RNA extraction. For these apoplastic conditions, two RNA samples were obtained each biological replicate, with each RNA sample including samples from 40–80 leaves. RNA samples were treated with TURBO^™^ DNase (Ambion, Austin, TX) to remove residual DNA. The RNA was tested for DNA contamination using RT-PCR where RNA is used as the template with no reverse transcription reaction. The RNA quality and quantification was evaluated utilizing an Agilent 2100 Bioanlyzer (Agilent Technologies, Inc.), selecting samples with RNA Integrity Number (RIN) above 8.0 [32].

For qRT-PCR analysis, selected RNA samples were converted to cDNA by reverse transcription using Super Script Vilo^™^ cDNA Synthesis kit (Invitrogen) as described by Greenwald et al. [32], and diluted to 10 ng/μl. Reverse transcription was performed with the following temperature cycle: 10 min at 25°C, 60 min at 42°C, and 5 min at 85°C.

### qRT-PCR analysis

An Applied Biosystems 7500 Fast Real-Time PCR System was used in conjunction with SYBR^®^ Select Master Mix (Invitrogen) for qRT-PCR analysis. For each 20 μl reaction the following was used: 10 μl SYBR^®^ Select Master Mix, 8.20 μl nuclease-free water, 0.4 μl of both the forward and reverse primers (200 nM), and 1 μl of template cDNA (10 ng/μl). Primers used for qRT-PCR analysis as listed in S2 Table along with primers specific for *recA* and *16s-rRNA* as internal control genes that were used to normalize gene expression [28]. For each primer pair, the linearity of detection was confirmed to have a correlation coefficient of at least 0.98 (r^2^>0.98) over the detection area by measuring a 5-fold dilution curve with cDNA generated from bacterial RNA. Conditions for qRT-PCR involved an incubation temperature of 95°C for two minutes, followed by 40 cycles involving 3 seconds at 95°C and 30 seconds at 60°C. A melting curve analysis was used for each qRT-PCR reaction to validate that a single primer product was amplified. qRT-PCR was performed to determine the effects of apoplastic and HMM conditions on the expression of genes that encode LuxR-like proteins in parental strain *Pss* B728a. The expression of genes associated with syringomycin biosynthesis, epiphytic fitness and secondary metabolism was also determined in parental strain *Pss* B728a compared to *salA*, *syrF*, and *syrG* deletion mutants in HMM media conditions.

Data was analyzed using the comparative C_t_ method [46], where an increase or decrease of transcript levels is determined by comparing the C_t_ values of the samples of interest to the C_t_ values of a control sample. Fold change in gene expression was calculated using the following equation: 2^-ΔΔCt^ = (C_t gene-of-interest_−C_t internal control_) Treated sample–(C_t gene-of-interest_−C_t internal control_) Untreated sample [46]. A 2-fold or more change in C_t_ for the sample of interest when compared to the control sample was considered to be significant [36]. A decrease in fold change was computed by taking the negative inverse of the fold change value [46].

### Operon analysis of *salA*, *syrF*, and *syrG* in *Pss* B728a using RT-PCR

RT-PCR analysis was performed to define the operons that encompass *salA*, *syrF*, and *syrG* using RNA isolated from *Pss* B728a. Primers designed for *salA*, *syrF*, *syrG*, and neighboring genes (S1 Table) were used to identify if they were transcribed as monocistronic or polycistronic mRNA. Total RNA was prepared from *Pss* B728a by growing cells on HMM agar for 48 h at 26°C and harvesting total RNA using an RNeasy Mini Kit along with the RNAprotect reagent following the manufacturer’s protocol (Qiagen Inc., Valencia, CA). RNA samples were treated with TURBO^™^ DNase (Ambion, Austin, TX) to remove residual DNA. The RNA was tested for DNA contamination using RT-PCR where RNA is used as the template with no reverse transcription reaction. The RNA quality and quantification was evaluated using an Agilent 2100 Bioanlyzer (Agilent Technologies, Inc.), and selecting samples with an RNA Integrity Number (RIN) above 8.0 [32].

Using approximately 100 ng of total RNA from *Pss* B728a, RT-PCR was performed using an Applied Biosystems 7500 Fast Real-Time PCR System with a OneStep RT-PCR kit (Qiagen Inc., Valencia, CA) according to the manufacturer’s instructions. Reverse transcription was performed by incubating at 50°C for 30 min. After reverse transcription, RT-PCR was carried out using the following temperature cycle: 95°C for 15 minutes, followed by 30 cycles involving 30 seconds at 94°C and 30 seconds at 55°C. After RT-PCR, amplified products were subjected to electrophoresis.

### Primer extension analysis

Primer extension was performed using the Primer Extension System (Promega, Madison, WI), and a sequence marker that was created using the Sequenase Version 2.0 DNA sequencing kit following the manufacturer’s instructions (Affymetrix, Santa Clara, CA). Oligonucleotides salAPE, syrFPE, and syrGPE were radiolabeled with [γ-^32^P]ATP (Perkin Elmer, Inc., Boston, MA) at the 5’ end. Primer extension was performed with 1.0 pmol of the labeled primer and 15 μg of total RNA from *Pss* B728a. Total RNA from *Pss* B728a was prepared as described previously [32]. The plasmids pLV2602, and pLV2607 were used as templates to create sequencing ladders of the upstream regions of *salA*, *syrF*, and *syrG*.

### Computer analysis

Nucleotide sequences that were 100-bp upstream of identified transcriptional start sites were analyzed using the Softberry Bprom algorithm (http://linux1.softberry.com/berry.phtml) to identify putative σ^70^-dependent promoters. These putative promoter sequences were aligned with T-Coffee [47].

### Construction of GFP translational fusions and mutagenesis

To define and characterize the promoter regions of *syrF* and *syrG*, promoter fragments were PCR amplified from *Pss* B728a genomic DNA using primers listed in S1 Table and cloned into a *gfp* broad-host-range promoter-probe vector, pPROBE-KT’, resulting in translational fusions to *gfp*. *Bam*HI and *Sac*I restriction enzyme sites flanked all promoter fragments. Amplified promoter fragments were digested with *Bam*HI and *Sac*I along with the broad-host-range promoter-probe vector, pPROBE-KT’[39]. Digested PCR products and vector were purified using Wizard^®^ SV Gel and PCR Clean-Up System (Promega) and quantified utilizing micro-spectrophotometry (Nano-Drop Technologies, Inc.). Ligation of the vector and insert was performed using T4 DNA ligase (New England Biolabs) and chemically transformed into *E*. *coli* One Shot^®^ TOP10 cells (Invitrogen) for confirmation of constructs. Additionally, *salA*, *syrB1*, and *syrP* promoters were cloned in pPROBE-KT’. Cloned pPROBE-KT’ constructs were introduced into B728a derivative mutants by electroporation.

### GFP assays

Quantitative GFP assays were performed as described by Miller et al. [39]. *E*. *coli* cells were cultured overnight in LB with the appropriate antibiotics at 37°C with shaking. For *Pss* B728a, cells were cultured overnight in NBY with the appropriate antibiotics at 26°C with shaking. Cells were harvested, washed, and resuspended in 10 mM phosphate buffer to a concentration of 2 x 10^9^ cells per mL. GFP fluorescence was measured on Tecan SpectraFluor (Tecan) at an excitation wavelength of 485 nm, and an emission wavelength of 525 nm. Intensity readings were represented by arbitrary units and normalized to a cell density of 10^9^ cells per mL.

## Results

### SalA, SyrF, and SyrG are novel LuxR transcriptional regulators with homologs found exclusively in *Pseudomonas syringae* genomospecies 2

An investigation of the *Pss* B728a genome identified 24 genes encoding LuxR-like regulatory proteins, which were also found in the genome of *Pss* B301D, displaying a high degree of nucleotide conservation between these two bacterial strains. Three genes of interest are *salA*, *syrG*, and *syrF* that are located adjacent to the syringomycin gene cluster. A conserved domain search of *salA*, *syrG*, and *syrF* using NCBI Conserved Domains database (http://www.ncbi.nlm.nih.gov/Structure/cdd/) confirmed the presence of a conserved helix-turn-helix DNA binding motif. The HTH DNA binding motif on the C-terminal region of the proteins is typical of LuxR regulatory proteins, but they lacked an N-terminal autoinducer-binding domain and receiver domain. BLAST analysis was also performed on SalA, SyrF, and SyrG protein sequences to determine the degree of conservation in pseudomonad strains. It was observed that all three regulatory proteins are exclusively found in *P*. *syringae* genomospecies 2, with the C-terminal region being highly conserved with regulatory protein SyrG (S1 Fig).

### Expression of the *syrG* and *syrF* genes in the apoplast of bean relative to HMM medium

**Fig 2:**
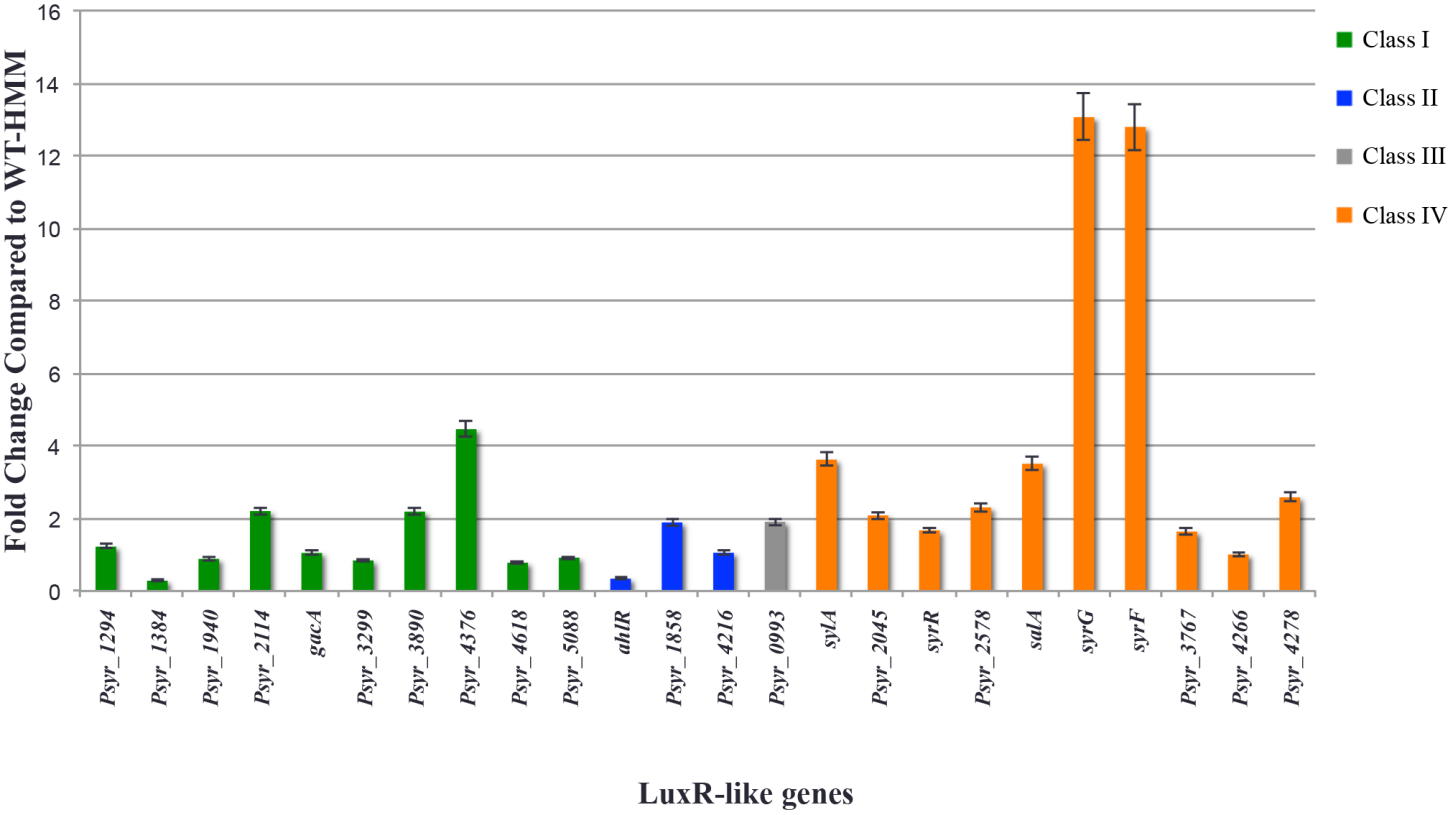
Expression analysis in the apoplast of bean of genes encoding LuxR-like proteins in *Pss* B728a. The genes that encode proteins that are classified in the first subfamily of LuxR (Class I) are shown in the green and are typically associated with two component signal transduction systems. Shown in blue are genes that encode LuxR-like proteins implicated in quorum sensing based on domain architecture. Psyr_0993, which is shown as gray, is the only gene that encodes a protein characterized as a LAL or LuxR-like proteins that require ATP for activation. The final subfamily of LuxR-like proteins are encoded on genes shown in orange bars, which lack an N-terminal regulatory domain and are associated with secondary metabolism. Out of all 24 LuxR-like proteins found in the genome of *Pss* B728a, the genes encoding SyrG and SyrF are the most highly expressed in the apoplast when compared to HMM liquid medium. The values are represented as the average fold change of three technical replicates of three biological samples. Gene expression was normalized to the *16s-rRNA* and *recA* internal control genes. Vertical bars indicate standard errors of the average values over triplicate runs.

Analysis of the 6.09 Mb *Pss* B728a genome identified 24 genes dispersed throughout the genome ecoding LuxR-like proteins. Some of these proteins have been implicated in virulence and secondary metabolism in *Pss* B728a. To identify the LuxR-like proteins that are important to the plant-pathogen interaction, qRT-PCR analysis was used to determine transcript abundance of the genes identified as LuxR-like proteins in the apoplast of bean when compared to parental strain B728a in conditions conducive for *hrp* gene expression. The results indicated that in *Pss* B728a, change in gene expression between these conditions was the greatest for both *syrG* and *syrF* genes in the apoplast of bean relative to HMM liquid medium (Fig 2). This result is consistent with earlier reports of *syrG* gene expression levels in the apoplast [5, 10]. Higher expression levels of these genes in the apoplast is attributed to the presence of plant signal molecules known to activate expression of *syr* genes [6]. The relative expression of *syrG* and *syrF* when compared to other *luxR* genes indicate that the SyrG and SyrF proteins are involved in the transcriptional regulation of genes that potentially are critical to plant pathogenesis.

### SalA, SyrF, and SyrG influence virulence of *Pss* B728a on bean plants

**Fig 3:**
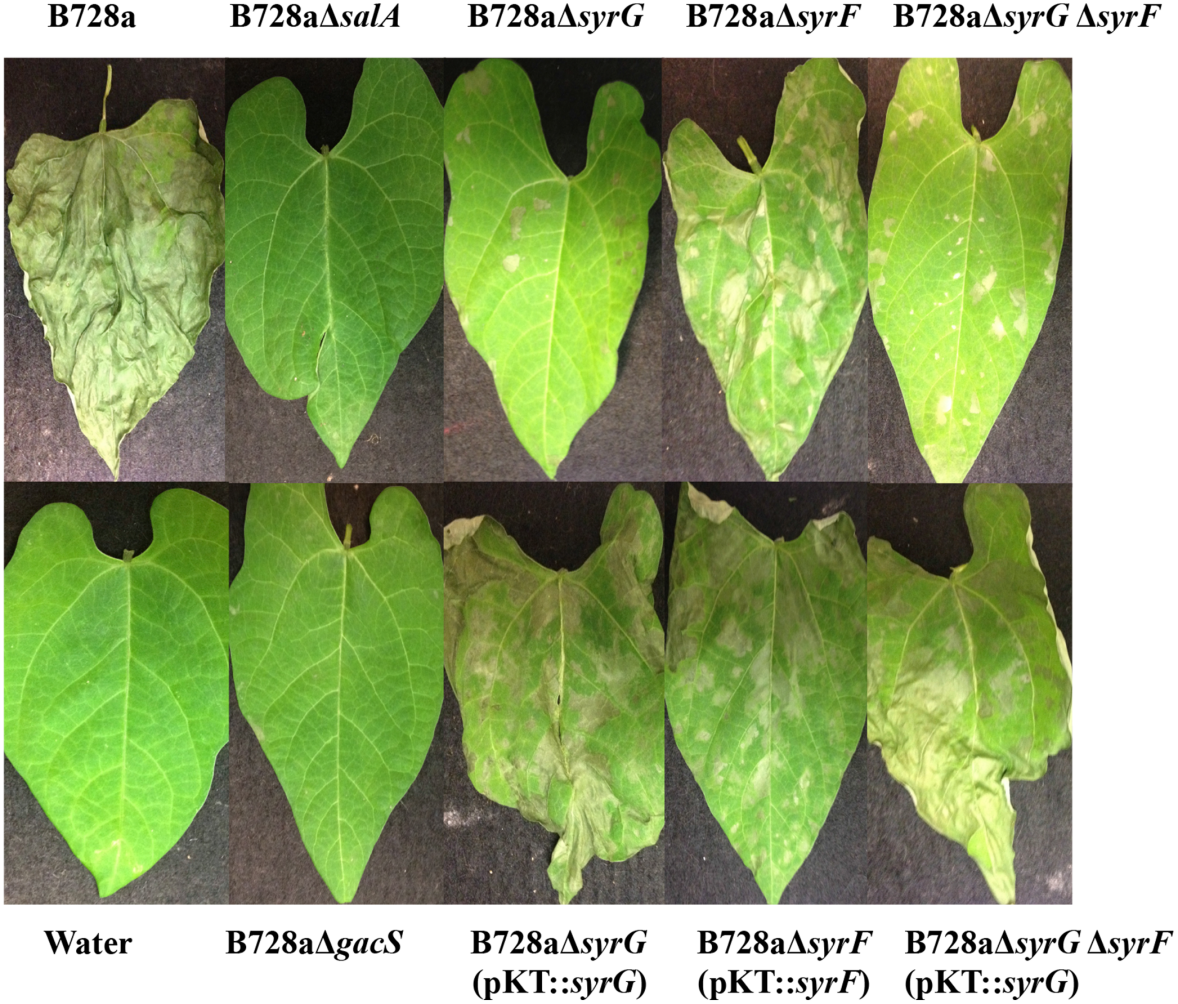
Pathogenicity assays to evaluate the contribution of *syrG* and *syrF* to virulence on bean. Bean leaves were inoculated by vacuum infiltration with bacterial suspensions containing 10^7^ CFU/cm^2^ of either B728a, B728aΔ*syrG*, B728aΔ*syrF*, B728aΔ*syrGΔsyrF*, B728aΔ*gacS*, B728aΔ*syrG* (pKT::*syrG*), B728aΔ*syrF* (pKT::*syrF*), or B728aΔ*syrG*Δ*syrF* (pKT::*syrG*). Plants were maintained at room temperature in a growth chamber for 72 h. Necrotic lesion surface areas were calculated using ImageJ software. This experiment was performed in triplicate, and representative results are shown.

The generation of a clean deletion mutant of *syrG* and *syrF* in *Pss* B728a was achieved by using the mutation strategy described in the Materials and Methods. The bacterial strains B728aΔ*syrG*, B728aΔ*syrF*, and B728aΔ*syrG*Δ*syrF* displayed colony morphologies and growth curve patterns similar to parental strain B728a (data not shown). The derivative mutants B728aΔ*syrG*, B728aΔ*syrF*, and B728aΔ*syrG*Δ*syrF* were significantly reduced in virulence relative to the parental strain *Pss* B728a shown in Fig 3. The *salA* mutant failed to produce watersoaked necrotic lesions typical of *Pss* B728a. The *salA* mutant was comparable to B728aΔ*gacS* in regards to virulence by lacking the ability to produce necrotic lesions and cause disease on bean. B728aΔ*syrG* was reduced in virulence by approximately 95% when compared to the parental strain *Pss* B728a. Mutants of *syrG* displayed small, non-spreading lesions with the average surface area of 45.1 mm^2^ on bean leaves. In contrast, the *syrF* mutant was able to produce large necrotic lesions, but displayed approximately 61% reduction in virulence on bean with the average necrotic lesion surface area of 284.5 mm^2^. The double deletion mutant of *syrG* and *syrF* exhibited disease symptoms comparable to the *syrG* mutant. Virulence of *syrG* and *syrF* derivative mutants was partially restored *in trans* by complementation of *syrG* and *syrF* (Fig 3).

Bacterial populations of infected bean plants were monitored over a 3-day period. At 3 days post-inoculation, bacterial titers for parental strain *Pss* B728a was 6.5 x 10^7^ CFU/cm^2^, while B728aΔ*salA*, B728aΔ*syrG* and B728aΔ*syrF* were 2.0 x 10^6^ CFU/cm^2^, 3.2 x 10^7^ CFU/cm^2^ and 1.5 x 10^7^ CFU/cm^2^, respectively. B728aΔ*gacS*, which fails to produce disease on bean, maintained a population equivalent to day 0 of 2.2 x 10^4^ CFU/cm^2^ 3 days post-inoculation. The bacterial population of B728aΔ*syrG* and B728aΔ*syrF* were not significantly different from the parental strain *Pss* B728a, indicating that the *syrG* and *syrF* genes are not required for multiplication *in planta*. In contrast, B728aΔ*salA* displayed a 10-fold reduction in bacterial titers compared to parental strain B728a. B728aΔ*salA* is able to replicate *in planta*, but at a reduce rate when compared to B728a. In the case of B728aΔ*gacS*, the bacterium remains viable but is limited on its ability to multiply *in planta*.

### Deletion mutants of the *salA*, *syrF*, and *syrG* genes in *Pss* B728a affect syringomycin production

**Fig 4:**
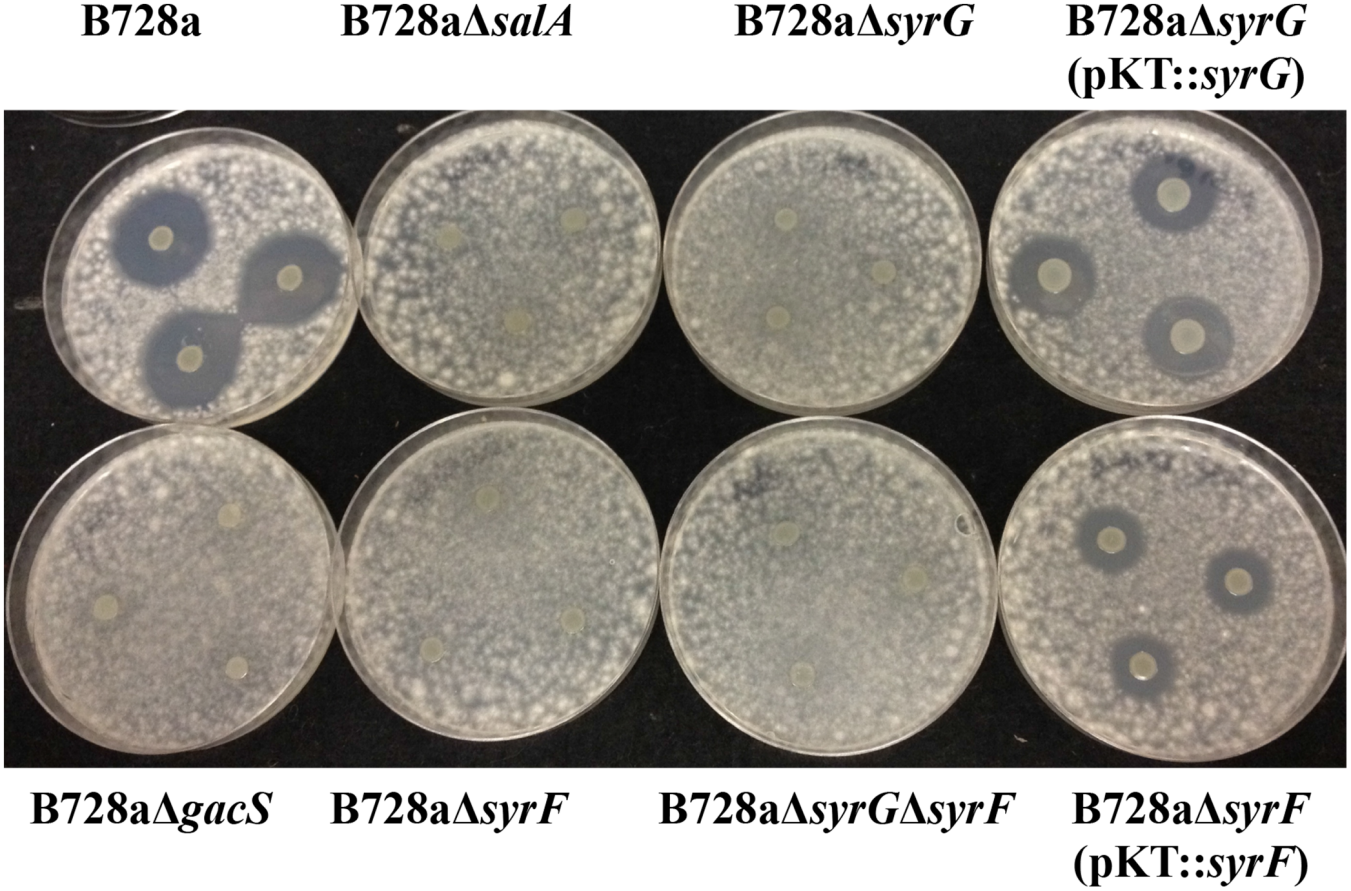
Bioassy to evaluate syringomycin production in parental strain B728a and derivative mutants. Bacterial strains were grown on HMM for 4 days. Plates were oversprayed with *Geotrichum candidum* and incubated 24 h at 26°C to observe zones of inhibition indicative of syringomycin production. The experiment was repeated in triplicate.

The bioassay used to evaluate the influence deletion of *salA*, *syrG*, and *syrF* has on syringomycin production was determined by measuring zones of antifungal activity to *G*. *candidum* as compared to parental strain *Pss* B728a grown on HMM agar (Fig 4). All the deletion mutants, including the double mutant *Pss* B728aΔ*syrF*Δ*syrG*, displayed no measurable antifungal activity toward *G*. *candidum*. Antifungal activity toward *G*. *candidum* was partially restored when B728a mutant derivatives were complemented *in trans* with the vector pPROBE-KT’ carrying an intact copy of the *syrF* or *syrG* gene. These results differed from a previous study by Lu et al. [10] with site directed insertional mutants of *salA*, *syrF*, and *syrG* in *Pss* B301D. It is hypothesized that the insertional mutants of *syrF* and *syrG* produce truncated proteins with reduced functional activity displaying low levels of toxin production. In contrast, clean deletion mutants of *syrF* and *syrG* were generated in *Pss* B301D. These derivative mutants displayed a loss of syringomycin production similar to the derivative mutants generated in *Pss* B728a (data not shown).

### Overexpression of N-terminal truncated proteins of SyrG and SyrF has an effect on syringomycin production

**Fig 5:**
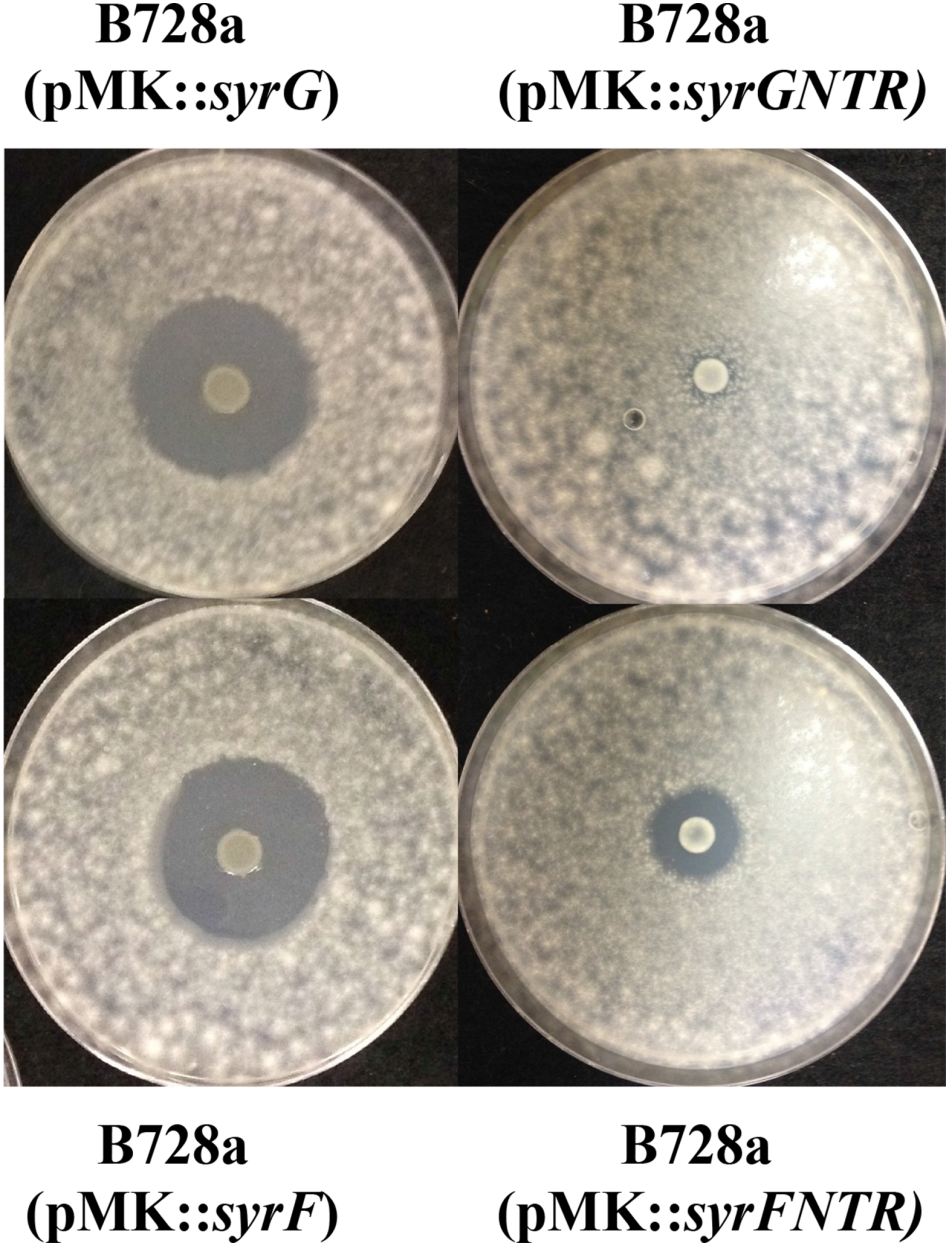
Effect of overexpression of N-terminal region (NTR) of SyrG and SyrF on syringomycin production in *Pss* B728a. Bacterial strains were grown on HMM for 4 days. Plates were oversprayed with *Geotrichum candidum* and incubated 24 h at 26°C to observe zones of inhibition indicative of syringomycin production. The experiment was repeated in triplicate.

To test the hypothesis that insertional mutants of *syrF* and *syrG* are leaky mutations and to demonstrate the HTH DNA binding domain of SyrG and SyrF are essential for binding to *syr-syp* promoters, the SyrG and SyrF proteins lacking the C-terminal HTH DNA-binding domain were overexpressed in *Pss* B728a. The overexpression of SyrG and SyrF had no effect on syringomycin production. However, overexpression of the N-terminal regions lacking the C-terminal HTH DNA-binding domains of SyrG and SyrF in *Pss* B728a resulted in a marked reduction of syringomycin zones of inhibition to *G*. *candidum* from 16 mm to 0.5 mm and 3 mm, respectively (Fig 5). The overexpression of the N-terminal regions of SyrG and and SyrF resulted in a 97% and 81% reduction in syringomycin production, which can be attributed to nonfunctional heterodimers formed between wild-type proteins and the truncated proteins. These nonfunctional heterodimers lack the ability to properly bind to *syr-syp* promoters, which is essential for the transcriptional activation of genes required for phytotoxin production. The truncation of SyrG displayed the greatest reduction in syringomycin production, which is comparable to the overexpression of an N-terminal truncated SalA in *Pss* B301D [12].

### The effect of *syrG* and *syrF* deletion mutants on genes associated with virulence

**Fig 6:**
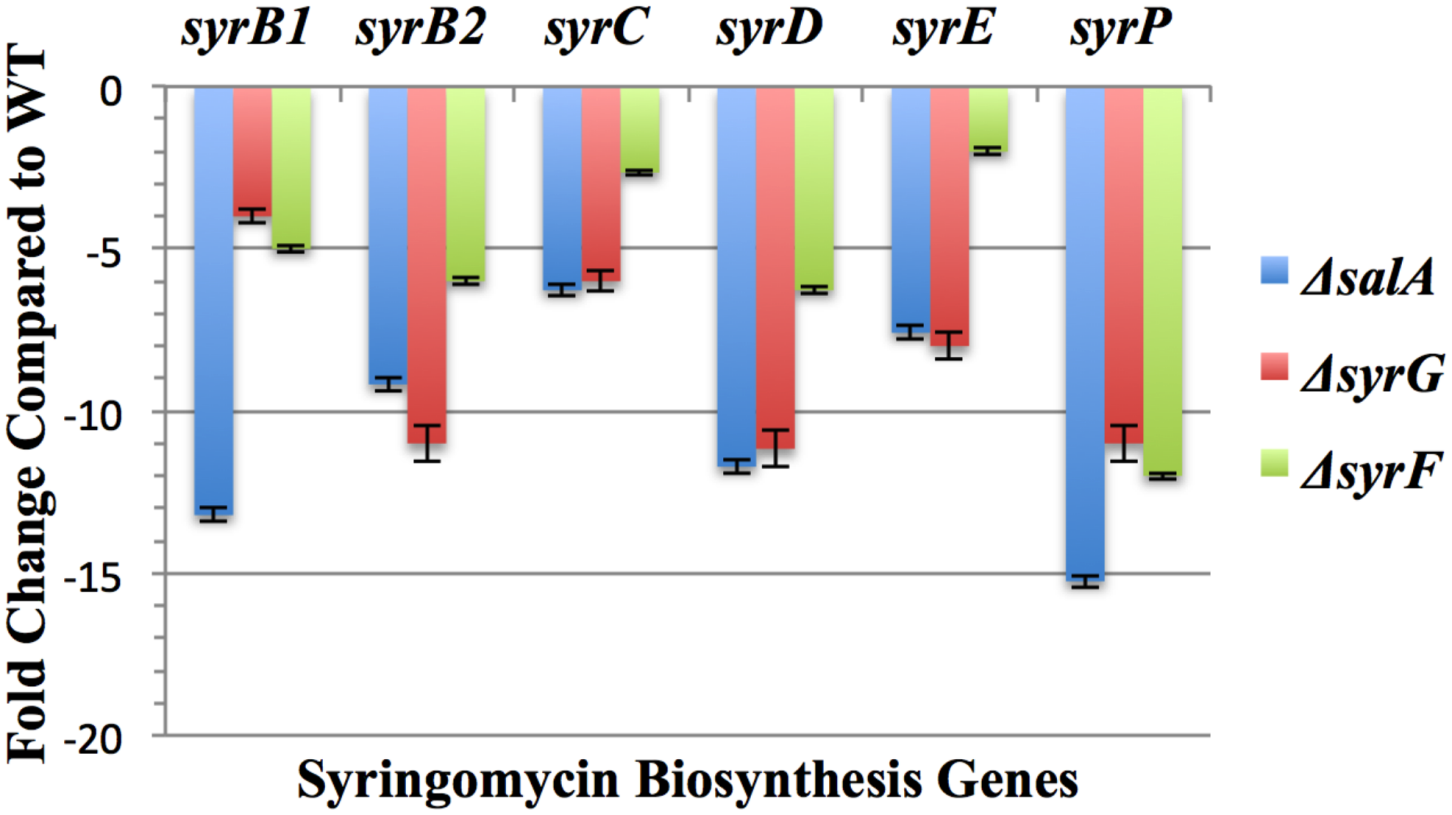
Quantitative real-time PCR analysis of syringomycin biosynthesis genes in Δ*salA*, Δ*syrG*, and Δ*syrF* mutants of *Pss* B728a. The values represent the average fold change in gene expression from parental strain *Pss* B728a; the results are the averages of three technical replicates from three biological samples grown in HMM liquid medium. Gene expression levels were normalized to *16s-rRNA* and *recA* internal control genes, and vertical bars indicate standard errors of the average values over triplicate runs. Negative values indicate a decrease in transcript abundance by taking the negative inverse of a fold change value less than 1.

Previous experiments have established both *syrG* and *syrF* have an influence on syringomycin production and virulence in *Pss* B728a. Quantitative real-time PCR [48] was used to identify the effect *syrG* and *syrF* deletion mutants have on genes associated with syringomycin production and virulence. A total of 23 genes were evaluated using qRT-PCR that included genes involved in the biosynthesis of syringomycin, syringopeptin, achromobactin, alginate, levansucrase, syringolin, syringafactin, and pyoverdine. Both *syrG* and *syrF* had an effect on the expression of syringomycin biosynthesis genes (Fig 6). A deletion mutant of *syrG* resulted in a 4- to 11-fold decrease in transcript abundance of *syrB1*, *syrB2*, *syrC*, *syrD*, *syrE* and *syrP*. In regards to *syrF*, the deletion mutant resulted in a 2- to 12-fold decrease of transcript abundance of syringomycin biosynthesis genes. The results also indicated that both SyrG and SyrF are involved in the transcriptional regulation of genes associated with syringomycin production. Mutants of *syrG* and *syrF* did not appear to have an effect on the expression of genes associated with achromobactin, alginate, levansucrase, syringolin, syringofactin, or pyoverdine biosynthesis (data not shown). Both *syrG* and *syrF* mutants failed to have an effect on known virulence genes outside of the *syr-syp* gene cluster.

### The effect of *syrF* and *syrG* deletion mutants on LuxR-like homologs in *Pss* B728a

**Fig 7:**
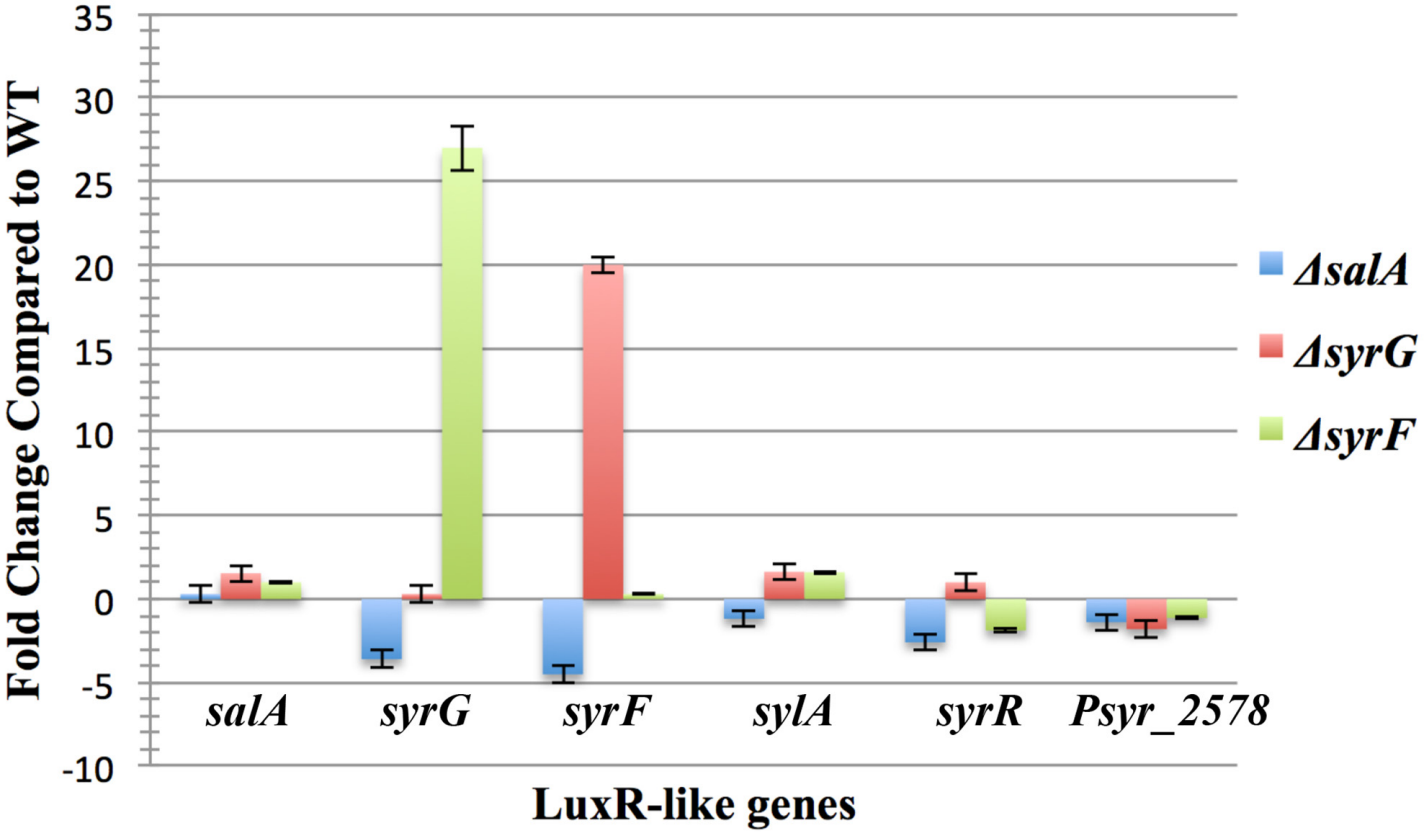
Quantitative real-time PCR analysis of LuxR-like genes in Δ*salA*, Δ*syrG*, and Δ*syrF* mutants of *Pss* B728a. The values represent the average fold change in gene expression from parental strain *Pss* B728a; the results are the averages of three technical replicates from three biological samples grown in HMM liquid medium. Gene expression levels were normalized to *16s-rRNA* and *recA* internal control genes, and vertical bars indicate standard errors of the average values over triplicate runs. Negative values indicate a decrease in transcript abundance by taking the negative inverse of a fold change value less than 1.

To determine the effect *syrF* and *syrG* mutants have on LuxR-like homologs in *Pss* B728a, qRT-PCR analysis was performed using primers specific for *salA*, *syrG*, *syrF*, *sylA*, *syrR*, and *Psyr_2578*. SylA and SyrR are LuxR-like proteins that have been implicated in the regulation of syringolin and syringofactin [5, 35, 49]. Results from qRT-PCR analysis show that *syrG* and *syrF* require a functional *salA* gene for activation (Fig 7). Mutants of *salA* displayed a 3.6-, and 4.5-fold decrease in transcript abundance of *syrG* and *syrF*, respectively. Mutants of *syrG* displayed a 27-fold increase in transcript abundance of *syrF*, and mutants of *syrF* displayed a 20-fold increase in transcript abundance of *syrG*. Data obtained from qRT-PCR analysis indicated that both *syrG* and *syrF* negatively regulate expression of each other’s gene. SyrG and SyrF did not have an effect on the expression of the LuxR-like genes *sylA*, *syrR*, and *Psyr_2578* indicating they are not part of the SyrG or SyrF regulatory networks.

### The *syrF* gene is in an operon with *oprM*, whereas *salA* and *syrG* are monocistronic mRNA transcripts

The *syr-syp* gene cluster consists of genes involved in the biosynthesis, regulation and secretion of syringomycin and syrinopeptin. These genes are organized into two operons that were defined by Wang et al. [50] in *Pss* B301D. Located adjacent to the *syr-syp* gene cluster are the LuxR-like regulatory genes *salA*, *syrF*, and *syrG*. RT-PCR analysis (data not shown) revealed that both *salA* and *syrG* are transcribed as moncistronic mRNA with their own native promoter regions. There were no *salA*-*syrG* products observed with RT-PCR using primers specific to the 3’ and 5’ sequences of these genes, respectively. The genes that are organized in a polycistronic operon are *syrF* and *oprM*. A *syrF-oprM* product was obtained with RT-PCR using primers oprMF and syrFR that were specific for the 3’ and 5’ sequences of these genes, respectively.

### Characterization of transcriptional start sites of the *syrG* and *syrF* genes

**Fig 8:**
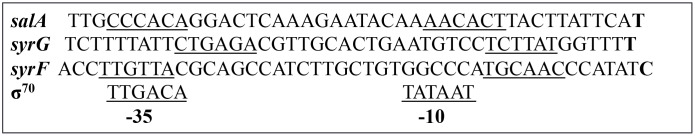
Comparison of putative promoter sequences of *salA*, *syrG* and *syrF*. Predicted promoter sequences are based on defined transcriptional start sites and using BPROM promoter prediction software. Conserved sequence motifs corresponding to -35 and -10 promoter regions are underlined and compared to a σ^70^ dependent promoter sequence. The defined promoter region of *salA* is distinctly different from the predicted promoter regions of *syrG* and *syrF*.

**Fig 9:**
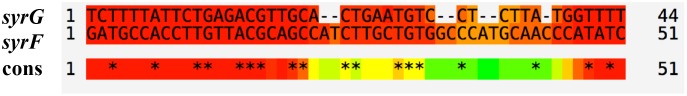
Alignment of *syrG* and *syrF* promoter sequences in *Pss* B728a. The predicted promoter sequences of *syrG* and *syrF* were aligned using T-COFFEE and conserved sites are shown as asterisks. The color code is based on CORE index, using consistency among pairwise alignments for estimating reliability. Sequences shown in red indicate high reliability, where green is indicative of low reliability.

After identifying the monocistronic and polycistronic transcripts of *salA*, *syrF*, and *syrG*, the transcriptional start sites of the respective genes were defined using primer extension analysis. For *salA*, the salAPE primer was radiolabeled and used to identify the transcriptional start site. Primer extension analysis revealed the transcription start site was at the thymine residue, which was 63 bp upstream to the translational start codon of *salA*. This result is similar to the transcriptional start site identified for *salA* in *Pss* B301D by Wang et al. [50]. However, the prior study in *Pss* B301D did not identify the transcriptional start sites for *syrF* and *syrG*. For *syrF*, the syrFPE primer was used to identify the transcriptional start site at the cytosine residue, which was revealed to be 498 bp upstream of the translation start codon. The syrGPE primer was used to identify the transcriptional start site at the thymine residue, 235 bp upstream of the translation start codon. The transcriptional start site of *syrG* suggests a putative promoter region sequence, CTGAGAN_17_TCTTAT (Fig 8). Similarly, the transcriptional start site of *syrF* suggests a putative promoter region sequence, TTGTTAN_23_TGCAAC. In addition, computer analysis of promoter sequences identified conserved sequences observed around the -35 promoter regions of *syrG* and *syrF*, illustrated by the nucleotide sequence alignment of the predicted promoter regions (Fig 9). These putative promoter regions were predicted using defined transcriptional start sites and BPROM promoter prediction software [51]. Both promoters share high similarity to the consensus promoter sequence of σ^70^ found in Gram-negative bacteria [52].

### Identification of essential *syrG* and *syrF* promoter regions

**Fig 10:**
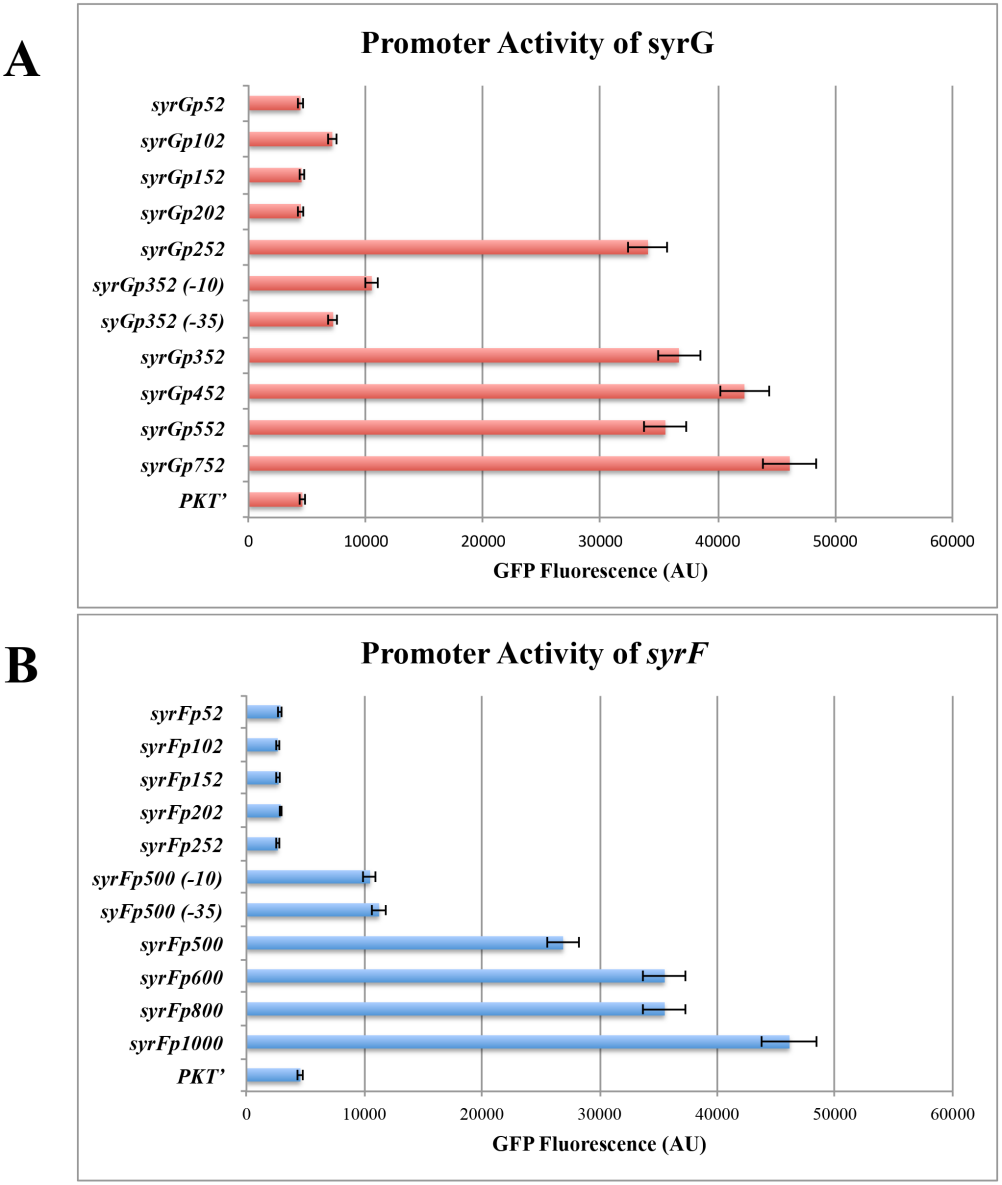
Analysis of the promoter regions of *syrG* (A) and *syrF* (B) by testing the effect deletion mutants have on the expression of *syrG*::*gfp* and *syrF*::*gfp* transcriptional fusions. All *gfp* constructs were electroporated into cells of *Pss* B728a to measure GFP fluorescence (AU). All measurements were averages over three technical replicates of three biological samples. Horizontal bars represent the standard error of the average values.

The effects of promoter deletion mutants on the expression of *syrG*::*gfp* and *syrF*::*gfp* fusions are shown in Fig 10. For *syrG*::*gfp* fusions, deletion constructs were generated from 752-bp, 552-bp, 452-bp, 352-bp, 252-bp, 202-bp, 152-bp, 102-bp, and 52-bp upstream of the translational start site of *syrG*. For *syrF*::*gfp* fusions, deletion constructs were generated from 1000-bp, 800-bp, 600-bp, 500-bp, 252-bp, 202-bp, 152-bp, 102-bp, and 52-bp upstream of the translational start site of *syrF*. Results indicated that the 100-bp region of 252- to 352-bp upstream of the start codon of *syrG* is critical for the expression of the *syrG*::*gfp* fusion. In addition, the 100-bp region of 500- to 600-bp upstream of the start codon of *syrF* is critical for the expression of the *syrF*::*gfp* fusion. When the predicted -10 promoter region TCTTAT of *syrG* and TGCAAC of *syrF* was substituted with CTGCAG, expression of the *syrG*::*gfp* and *syrF*::*gfp* reporters in *Pss* B728a decreased by 71% and 70%, respectively. When the predicted -35 promoter region CTGAGA of *syrG* and TTGTTA of *syrF* was substituted with CTGCAG, expression of the *syrG*::*gfp* and *syrF*::*gfp* reporters in *Pss* B728a decreased by 80% and 68%, respectively.

### LuxR-type transcription regulator SalA is a positive regulator of *syrF* and *syrG* promoters

The effects of deletion mutants of *salA*, *syrG*, and *syrF* have on *salA*::*gfp*, *syrG*::*gfp*, and *syrF*::*gfp* reporter gene activities are shown in Table 2. In the parental strain *Pss* B728a, the *salA*::*gfp* construct (pPKT::*salA*) displayed the relative GFP fluorescence of 43044 AU/10^9^ CFU. Fluorescence of a *salA*::*gfp* fusion was reduced by 76% in a *salA* derivative mutant of *Pss* B728a. Deletion mutants of *syrG* and *syrF* did not significantly reduce the GFP fluorescence of a *salA*::*gfp* transcriptional fusion. Reporter gene activities of *syrG*::*gfp* and *syrF*::*gfp* were significantly reduced by 91% and 95% in B728aΔ*salA* as compared to activities in *Pss* B728a. This indicates that SalA is a transcriptional activator that functions upstream of *syrG* and *syrF*.

**Table 2:** Effect of *salA*, *syrG*, and *syrF* mutants on *gfp* reporter gene activity for *salA*, *syrG*, *syrF*, and *syrB1*.^a^

**Strain (reporter fusion)**	**Gene**	**GFP fluorescence ± SE**
B728a (*salA*::*gfp*)	*salA*	43,044 ± 203
B728aΔ*salA* (*salA*::*gfp)*	*salA*	10,055 ± 357
B728aΔ*syrG* (*salA*::*gfp*)	*salA*	42,991 ± 425
B728aΔ*syrF* (*salA*::*gfp)*	*salA*	38,106 ± 454
B728a (*syrG*::*gfp*)	*syrG*	45,776 ± 773
B728aΔ*salA* (*syrG*::*gfp)*	*syrG*	4,175 ± 237
B728aΔ*syrG* (*syrG*::*gfp)*	*syrG*	6,121 ± 449
B728aΔ*syrF* (*syrG*::*gfp)*	*syrG*	47,875 ± 389
B728a (*syrF*::*gfp*)	*syrF*	44,857 ± 718
B728aΔ*salA* (*syrF*::*gfp)*	*syrF*	2,202 ± 223
B728aΔ*syrG* (*syrF*::*gfp)*	*syrF*	6,163 ± 239
B728aΔ*syrF* (*syrF*::*gfp)*	*syrF*	7,713 ± 281
B728a (*syrB1*::*gfp*)	*syrB1*	47,266 ± 2,158
B728aΔ*salA* (*syrB1*::*gfp)*	*syrB1*	9,653 ± 626
B728aΔ*syrG* (*syrB1*::*gfp)*	*syrB1*	24,347 ± 522
B728aΔ*syrF* (*syrB1*::*gfp)*	*syrB1*	29,458 ± 345

^a^ The pPROBE-KT’ vector was used to construct pPKT fusions of specific genes to the *gfp* reporter. GFP fluorescence were averaged over three technical replicates of three biological samples followed by the standard error of the averaged values.

### SyrG is a positive regulator of *syrF* and both *syrG* and *syrF* are involved in the expression of *syrB1*

The effect deletion mutants of *syrG* and *syrF* have on the *syrG*::*gfp* and *syrF*::*gfp* reporter gene activities is shown in Table 2. The parental strain *Pss* B728a harboring the *syrG*::*gfp* transcriptional fusion displays a relative GFP fluorescence of 45776 AU/10^9^ CFU. GFP fluorescence of *syrG*::*gfp* decreased to 6121 AU/10^9^ CFU in *syrG* derivative mutants of *Pss* B728a, where the GFP fluorescence *syrG*::*gfp* was not significantly reduced in *syrF* deletion mutants. These results indicated that SyrG is required to activate its own gene expression, but SyrF does not have an affect on the promoter activities of *syrG*. The parental strain *Pss* B728a harboring the *syrF*::*gfp* transcriptional fusion displayed a relative GFP fluorescence of 44857 AU/10^9^ CFU. The relative GFP fluorescence of *syrF*::*gfp* decreased to 6163 and 7713 AU/10^9^ CFU in deletion mutants of *syrG* and *syrF*, respectively. Both SyrG and SyrF are required for activation of the *syrF* gene.

The parental strain *Pss* B728a harboring the *syrB1*::*gfp* transcriptional fusion displays a relative GFP fluorescence of 47266 AU/10^9^ CFU (Table 2). GFP fluorescence of *syrB1*::*gfp* decreased to 9653, 24347, 29458 AU/10^9^ CFU in deletion mutants of *salA*, *syrG*, and *syrF*, respectively. Both SyrG and SyrF are involved in the expression of *syrB1*.

### Overexpression of SyrF restores syringomycin production in *syrG* deletion mutants of *Pss* B728a

The derivative mutants of *syrG* and *syrF* in *Pss* B728a displayed a significant loss of syringomycin production when compared to the parental strain *Pss* B728a. The overexpression of SyrF had the ability to partially restore syringomycin production in *syrG* derivative mutants. Syringomycin inhibition zones increase from 0 mm to 25 mm, resulting in a 25% increase in syringomycin production when compared to a *syrG* derivative mutant. However, the overexpression of SyrG failed to restore syringomycin production in *syrF* derivative mutants (data not shown). These results indicated that SyrG is an upstream activator of *syrF*.

## Discussion

The genome of *Pss* B728a is relatively large in size (6.09 Mb), and encodes 24 LuxR-like proteins. Homologs of these LuxR-like proteins were also found in the genome of *Pss* B301D displaying a high degree of sequence conservation compared to *Pss* B728a. These LuxR-like proteins have been associated with a variety of biological processes that includes quorum sensing, virulence, and secondary metabolism in *Pss* B728a [5, 10, 28, 29, 35, 36, 49, 53]. The superfamily of LuxR-like proteins may be categorizied into four subfamilies based on domain architecture and mechanism of regulatory activation shown in Fig 1. Located adjacent to the syringomycin gene cluster are *salA*, *syrG*, and *syrF*, which encode three LuxR-like proteins classified into a subfamily of LuxR proteins not fully characterized. These proteins lack a defined N-terminal regulatory domain, but possess a highly conserved C-terminal HTH DNA binding domain. The HTH DNA binding motif is known to interact with the promoter elements of targeted regulatory genes to induce or repress transcription [12, 49]. Sequence analysis of SalA, SyrG, and SyrF showed that these LuxR-like proteins are closely related to FixJ and NarL. Both FixJ and NarL are LuxR-like proteins belong to a subfamily of LuxR-like proteins that are part of a two componenet signal transduction system that requires phosphorylation of the N-terminal receiver domain for activation [14, 15, 27]. However, SalA, SyrG, and SyrF share the greatest sequence homology to the LuxR-like protein GerE that is involved in the regulation of spore formation in *B*. *subtilis* [27]. The GerE protein also lacks an N-terminal regulatory domain [27]. LuxR-like proteins that lack a N-terminal regulatory domain are part of a LuxR subfamily that is not completely defined that may act as transcriptional activators and repressors. Analysis of the crystal structure of GerE revealed that it is comprised of four alpha helices, of which the central pair forms a HTH DNA-binding motif in the C-terminal region of the protein [27]. LuxR-like proteins exhibiting a similar domain organization have been associated with secondary metabolism in *Pss* B728a. For example, SlyA activates the transcription of *slyB* and *sylC*, which are involved in the biosynthesis of syringolin [49]. *SyrR* and *Psyr_2578* encode two LuxR-like proteins that are located adjacent to *syfA* and *syfB*. Both *syfA* and *syfB* are required for biosynthesis of syringafactin [5, 35, 44].

The importance of SalA, SyrG, and SyrF in regards to pathogenicity on bean plants was demonstrated by qRT-PCR analysis and pathogenicity assays. Quantitative real-time PCR analysis revealed that both *syrG* and *syrF* genes are expressed at high levels in the apoplast of bean relative to HMM liquid medium. This result indicated that both SyrG and SyrF are involved in regulation of genes important to establishing the plant-pathogen interaction or pathogenesis. Consequently, pathogenicity assays showed that mutants of *salA*, *syrG*, and *syrF* displayed a significant reduction in virulence by approximately 100%, 95%, and 61%, respectively. Disease development as observed for a mutant of *syrG* was comparable to a mutant of *salA*, whereas a mutant of *syrF* produced larger necrotic lesions on bean. A double mutant of *syrF* and *syrG* in B728a displayed symptomology comparable to the mutant of *syrG*. This result indicated that *syrG* is involved in regulating a broader range of genes critical to plant pathogenesis, and that it acts upstream of the LuxR-like protein SyrF in a regulatory cascade. Mutants of *syrG* and *syrF* were able to produce bacterial populations similar to the parental strain B728a *in planta*, indicating that *syrG* and *syrF* are not required for replication in the apoplast.

Reduction in virulence observed for *syrG* and *syrF* mutants can be attributed largely to the reduction in syringomycin and syringopeptin production. Syringomycin is considered one of the major virulence determinants of *Pss* B728a, along with syringopeptin [45]. Both *syrG* and *syrF* are required for syringomycin production (shown in Fig 4). Syringomycin production was partially restored when a functional copy of *syrG* and *syrF* was expressed *in trans*. Quantitative real-time PCR analysis also showed that mutants of *syrG* and *syrF* resulted in a significant decrease in the expression of syringomycin biosynthesis genes. These results were surprising given that a previous study by Lu et al. [10] using site-directed insertional mutants of *salA*, *syrF*, and *syrG* in *Pss* B301D, which displayed reductions of 100%, 83%, and 40% in syringomycin production, respectively, as compared to the parental strain. In contrast, clean deletion mutants of *syrF* and *syrG* were generated in *Pss* B301D; these derivative mutants corresponded to *syrF* and *syrG* mutants of *Pss* B728a with comparable reductions in syringomycin production (data not shown). It was hypothesized that the insertional mutants of *syrF* and *syrG* in *Pss* B301D produce truncated proteins with reduced functional activity as displayed by low levels of toxin production. This hypothesis was tested by overexpressing of the N-terminal regions of SyrG and SyrF in *Pss* B728a, which resulted in a significant reduction of syringomycin production (Fig 5). The overexpression of the N-terminal regions of SyrG and and SyrF resulted in 97% and 81% reduction in syringomycin production, which can be attributed to the formation of a nonfunctional heterodimers unable to bind to the promoter regions of genes associated with syringomycin production. Similar results were seen in *V*. *fischeri* when the overexpression of the N-terminal domain of LuxR displayed a reduction in luminescence [54]. The amino acids between 116 and 161 in the N-terminal domain were critical for LuxR to form dimers and activate transcription of the *luxICDABE* operon [54]. The truncation of SyrG displayed the greatest reduction in syringomycin production, which was comparable to the overexpression of the N-terminal region of SalA in B301D [12]. Wang et al. [12] showed that the overexpression of the N-terminal region of SalA and SyrF resulted in a significant decrease in expression of *syrB1*:*uidA* and *sypA*:*uidA* reporters. These results indicated that SyrG regulates a broader range of genes involved in toxin production as compared to SyrF.

Quantitative real-time PCR analysis was used to identify new components of the SyrG and SyrF regulons that had not been identified previously. The *syrG* and *syrF* regulatory genes do not appear to affect expression of genes involved in the production of alginate, achromabactin, levansucrase, syringolin, syringafactin, and pyoverdine. These results are not suprising given that a microarray study performed by Wang et al. [12] showed that genes involved in siderophore production, environmental stress, quorum sensing, global regulation, phytohormone synthesis and alginate production were not part the SyrF regulon. Both *syrG* and *syrF* mutants failed to have an effect on known virulence genes outside of the *syr-syp* gene cluster. But it seems that SyrG and SyrF negatively regulate the expression of each other’s gene to illustrate the complexity of their regulatory roles. Accordingly, it was established that both the SyrG and SyrF regulons overlap, and appear to be in competition for the binding and transcriptional activation of genes associated with syringomycin production.

It was revealed that the promoter sequences of *syrG* and *syrF* were highly similar to each other, but were distinctly different from the promoter region of *salA*. Alignment of these promoter sequences also identified a conserved sequence observed around the -35 region of the promoter. It was hypothesized that these conserved sequences are the binding site for SalA. Previous studies by Lu et al. [10] showed that SalA is required for the functional activation of *syrG* and *syrF*. In addition, Wang et al. [50] established that SalA binds to the promoter region of SyrF to activate transcription. It is unknown if similar conserved sequences observed around the -35 promoter region of *syrG* and *syrF* are found in the promoter region of *sylA*, given that *sylA* is under the transcriptional control of SalA [49]. It has been shown that the promoter of related genes, under the control of a LuxR regulatory protein, display conserved sites for binding of sigma factors and/or transcriptional regulators [50, 55, 56]. Wang et al. [50] identified a conserved 20-bp *syr-syp* box sequence around the -35 region promoter region in both syringomycin and syringopeptin biosynthesis genes. This *syr-syp* box was not identified in the promoters of *syrG* and *syrF*, which indicates that SalA has a promoter binding site sequence dissimilar from the *syr-syp* box to coordinate expression of the *syrG* and *syrF* regulatory genes. It was established that SyrF forms dimers that recognize the *syr-syp* box as a binding site [12, 50], but it remains unknown if SyrG recognizes the *syr-syp* box as a putative binding site. Similar binding sites occur in promoters of genes regulated by LuxRs, such as the *las-rhl* box identified in the promoters of genes controlled by quorum sensing in *P*. *aeruginosa* [49].

GFP reporter assays were used to determine the functional activity of promoters transcriptionally fused to *gfp* in *Pss* B728a mutant derivatives. A similar study by Ramel et al. [49] showed that the promoters of syringolin biosynthesis genes required *sylA* for activation. Our GFP assays showed that SalA is an upstream transcriptional activator of *syrG* and *syrF*. In addition, results demonstrated that SyrG is located upstream of SyrF in a regulatory cascade. Consequently, overexpression of *syrF* was able to restore syringomycin production in *syrG* derivative mutants, whereas overexpression of *syrG* was not able to restore syringomycin production in *syrF* derivative mutants. Thus, the regulatory effect of SyrG on the promoters of syringomycin biosynthesis genes may be indirect, which is consistent with the report of Wang et al. [12] that SyrF binds to the promoter of *syrB1*.

Characterization of the *salA*, *syrG*, and *syrF* genes is important to define the complex regulatory mechanism used for the expression of genes associated with virulence in *Pss* B728a. Based on previous studies [10, 12, 50] it was speculated that SyrG was responsible for the transcriptional activation of a broader range of genes associated with virulence than SyrF. However, this study did not identify virulence genes outside of the *syr-syp* gene cluster under the transcriptional control of SyrG. Nevertheless, this study demonstrated that SyrG is important for the transcriptional regulation of syringomycin and may be involved in an overlapping regulon with SyrF to control phytotoxin production. At the very least, SyrG appears to function as an upstream transcriptional activator of *syrF*. In conclusion, despite the complexity of the interactions between the SalA, SyrG, and SyrF regulators, they exhibit significant roles in toxigenesis during plant pathogenesis.

## Supporting Information

S1 Fig Sequence conservation of the SyrG regulatory protein in *Pseudomonas*. SyrG is conserved only in *Pseudomonas syringae* genomospecies 2 and not in *P*. *syringae* pv. *tomato* DC3000 genomospecies 1. Strains include: *P*. *syringae* pv. *syringae* (*Pss*) strains B301D, HS191, B728a, 642, and FF5, *P*. *syringae* pv. *japonica* M301072; *P*. *syringae* pv. *aptata* DSM 50252, *P*. *syringae* pv. *aceris* M302273, and *P*. *syringae* strain Cit7. There is also a high level of conservation observed in the C-terminal region of the protein where there is a HTH DNA binding motif known to interact with the promoter regions of targeted genes. The sequence of SyrG in *Pss* B301D, a closely related strain to *Pss* B728a, is one amino acid different from the sequence in *Pss* B728a.
(PDF)

S1 Table Primers used for PCR amplification and primer extension analysis. (PDF)

S2 Table Primers used for qRT-PCR analysis. (PDF)
