# Supplementary material for: Characterization of salA, syrF, and syrG Genes and Attendant Regulatory Networks Involved in Plant Pathogenesis by Pseudomonas syringae pv. syringae B728a
Source: PLoS One. 2016 Mar 8;11(3):e0150234. doi: 10.1371/journal.pone.0150234 (PMC4783005; doi:10.1371/journal.pone.0150234)
Supplement: S1 Fig — SyrG is conserved only in Pseudomonas syringae genomospecies 2 and not in P. syringae pv. tomato DC3000 genomospecies 1. Strains include: P. syringae pv. syringae (Pss) strains B301D, HS191, B728a, 642, and FF5, P. syringae pv. japonica M301072; P. syringae pv. aptata DSM 50252, P. syringae pv. aceris M302273, and P. syringae strain Cit7. There is also a high level of conservation observed in the C-terminal region of the protein where there is a HTH DNA binding motif known to interact with the promoter regions of targeted genes. The sequence of SyrG in Pss B301D, a closely related strain to Pss B728a, is one amino acid different from the sequence in Pss B728a. (PDF) [file pone.0150234.s001.pdf]

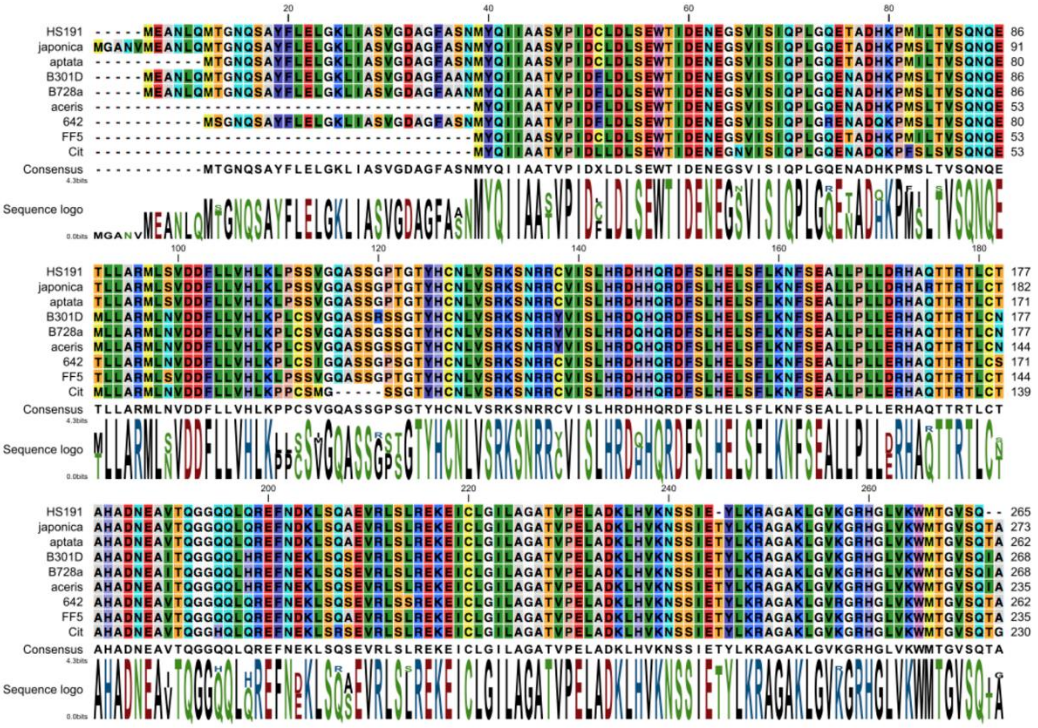

S1 Fig. Sequence conservation of the SyrG regulatory protein in *Pseudomonas*. SyrG is conserved only in *Pseudomonas syringae* genomospecies 2 and not in *P. syringae* pv. *tomato* DC3000 genomospecies 1. Strains include: *P. syringae* pv. *syringae* (*Pss*) strains B301D, HS191, B728a, 642, and FF5, *P. syringae* pv. *japonica* M301072; *P. syringae* pv. *aptata* DSM 50252, *P. syringae* pv. *aceris* M302273, and *P. syringae* strain Cit7. There is also a high level of conservation observed in the C-terminal region of the protein where there is a HTH DNA binding motif known to interact with the promoter regions of targeted genes. The sequence of SyrG in *Pss* B301D, a closely related strain to *Pss* B728a, is one amino acid different from the sequence in *Pss* B728a.
