# Supplementary material for: Characterization of salA, syrF, and syrG Genes and Attendant Regulatory Networks Involved in Plant Pathogenesis by Pseudomonas syringae pv. syringae B728a
Source: PLoS One. 2016 Mar 8;11(3):e0150234. doi: 10.1371/journal.pone.0150234 (PMC4783005; doi:10.1371/journal.pone.0150234)
Supplement: S1 Table — (PDF) [file pone.0150234.s002.pdf]

S1 Table. Primers used for PCR amplification and primer extension analysis.

Primer Name	Sequence (5' – 3')	Source
pE2602F	CACCGAACAGCCTTGTGCAGCGAG	This study
pE2602R	GTGAAACATTAACGTGCTCC	This study
pE2607F	CACCACCAGGTAGGTCGCAATGAC	This study
pE2607R	GTGGTGACGACCAAGGTTCT	This study
pLV2602-3F	CCGCGGGCCAGCCTCCACCGCAGAGCGTTG	This study
pLV2602-5R	GTGCACTACACATTTGCCCCCATGGCGTTA	This study
pLV2607-3F	GAGCTCACAGATCAATTGGCCCCTGGCTGT	This study
pLV2607-5R	CTCGAGATGTCCATTCCTGAAGATCAGAAG	This study
pLV2602KmF	CAAATGTGTAGTGCACGTGTAGGCTGGAGCTGCTTCG AAG	This study
pLV2602KmR	GGAGGCTGGCCCGCGGATTCCGGGGATCCGTCGACCT GCA	This study
pLV2607KmF	GAATGGACATCTCGAGGTGTAGGCTGGAGCTGCTTCG AAG	This study
pLV2607KmR	ATTGATCTGTGAGCTCATTCCGGGGATCCGTCGACCTG CA	This study
2602F	TCGGCTGGAGACTAACGCCA	This study
2602R	GCTCTGCGGTGGAGGCTGGC	This study
2607F	ATTGCACGCAACTTCTGATCT	This study
2607R	CCAGGGGCCAATTGATCTGT	This study
pKT2602F	GAATTCGAGCTCGCTCACGGTATTCCCCGCTCAATG	This study
pKT2602R	GTCGACGGATCCTCACGCTATTTGAGATACGCCTGT	This study
pKT2607F	GAATTCGAGCTCTCGCCCTCGCCAACCTCTGGAAAG	This study
pKT2607R	GTCGACGGATCCTCATTCTGCGCCTATCATCCATTT	This study
pKT-salApF	GAATTCGAGCTCGCCAGCCTCCACCGCAGAGCGTTG	This study
pKT-salApR	GTCGACGGATCCTCAGACAGCTGCCTGAAACATCTC	This study
pKT-syrB1pF	GAATTCGAGCTCGAACAAACTCCTGGACCTCAGCCC	This study
pKT-syrB1pR	GTCGACGGATCCTCAGACCGCTTCGAATTTCTTGCC	This study
pKT-syrG752pF	GAATTCGAGCTCGCTCACGGTATTCCCCGCTCAATG	This study
pKT-syrG552pF	GAATTCGAGCTCGGGGCGTCGCCGATTGCTCTATCC	This study
pKT-syrG352pF	GAATTCGAGCTCTCATGTATGTCGCTGTAAACGTCG	This study
pKT-syrG252pF	GAATTCGAGCTCATGTCCTCTTATGGTTTTTGCCAA	This study
pKT-syrG210pF	GAATTCGAGCTCGGGCGCTGGCGAACCGGCCTGTAG	This study
pKT-syrG202pF	GAATTCGAGCTCGCGAACCGGCCTGTAGCGAGACGT	This study
pKT-syrG102pF	GAATTCGAGCTCCAATCAGACAGTATACTCATTACT	This study
pKT-syrGR	GTCGACGGATCCTCACGCTATTTGAGATACGCCTGT	This study
pKT-syrF1300pF	GAATTCGAGCTCTCGCCCTCGCCAACCTCTGGAAAG	This study
pKT-syrFR	GTCGACGGATCCTCATTCTGCGCCTATCATCCATTT	This study
pKT-syrPpF	GAATTCGAGCTCGACCAAAGCTCCTGTGTAATAACC	This study
pKT-syrPpR	GTCGACGGATCCTCAGGCCGGTTGCCAAACGTCGCC	This study
pMK2602F	GTCGACGGATCCATGGAAGCCAATCTACAAATGACG	This study
pMK2602R	GTCGACTCTAGATCACGCTATTTGAGATACGCCTGT	This study
pMK2602-583R	GTCGACTCTAGACTGGCTCAGCTTTTCGTTGAATTC	This study

pMK2607F	GTCGACGGATCCATGAACCGACAAGTGAATGCCAAA	This study
pMK2607R	GTCGACTCTAGATCATTCTGCGCCTATCATCCATTT	This study
pMK2607-583R	GTCGACTCTAGAGTACTTGTACTCAATGGGAACCGA	This study
salAPE	ATGCGGGAAAAGCTGTTGCATGTT	This study
syrFPE	ATCTTTGGCATTCACTTGTCGGTT	This study
syrGPE	TCCCGTCATTTGTAGATTGGCTTC	This study
