# Supplementary material for: Characterization of salA, syrF, and syrG Genes and Attendant Regulatory Networks Involved in Plant Pathogenesis by Pseudomonas syringae pv. syringae B728a
Source: PLoS One. 2016 Mar 8;11(3):e0150234. doi: 10.1371/journal.pone.0150234 (PMC4783005; doi:10.1371/journal.pone.0150234)
Supplement: S2 Table — (PDF) [file pone.0150234.s003.pdf]

S2 Table. Primers used for qRT-PCR analysis.

Primer Name	Sequence (5' – 3')	Source
16sF	CTTCGGTACGCCTGGACA	[36]
16sR	CTTCGGTACGCCTGGACA	[36]
2578F	AACATGTCAATGCCCTGCAATCGG	This study
2578R	ACGGTATTGACCTTGAGTGCGAGT	This study
3983F	CGCTGGTATCGACACCTTTAT	This study
3983R	CAGTGGAACGACTGACTGATAG	This study
acsAF	ATTTGAATGCATCCTTTCCGGCCC	This study
acsAR	CTGATTGAAATCAAGCCGCTGGCA	This study
acsBF	TGCCGGGTCTGGACATGATTCTTG	This study
acsBR	ATTACCAGCGGGCAGCGTCTC	This study
acsCF	GTTTGACGTGTTTGCGCCATCGTA	This study
acsCR	TCATTGCGGCCTGTATGTTTGCAG	This study
acsDF	GAATTCGCCAGTCGTTGCTCTAC	This study
acsDR	GCTCGACGCGCTGCCACATC	This study
algAF	GTTATCTTGTCAGGCGGTAGTG	This study
algAR	TCAACGCCAGGAACTGTTTAG	This study
hrpAF	AGGGCATCAACAGCGTAAA	This study
hrpAR	ATCGCCTTTGCTGATGCT	This study
hrpEF	TGCTTGCCAAACGCAGTATTACC	This study
hrpER	TGACGTTGCGCGTCAGTCAGAATA	This study
hrpLF	CTATCAGGAAAGCTGGGAAGAC	This study
hrpLR	GCAATCGATGGCCTCTATGA	This study
hrpZF	TCCTGAAACCGAGACGACTGG	[36]
hrpZR	GACCGTTGCGCATCAGTTCCTC	[36]
lsc-1F	ACTGGTGAAGAGTGGGAAATAC	This study
lsc-1R	TTGCCGTCCTGAAAGACATAG	This study
oprMF	CGGTGACCAGCGGTACTTAT	This study
oprMR	ACTCGCCACCAGGCTTAGTA	This study
pslDF	GTTTACCGCCAACTCCATCTA	This study
pslDR	CCTTGACGGTGCCGATATAA	This study
pvdLF	CACCTACGCACTGGATGAAA	This study
pvdLR	AGTGATCAGTGGCCAGAAAC	This study
pvdSF	GCCGGAAATCTCGCATATCA	This study
pvdSR	CGGTACATCTCGAACGCATAA	This study
recAF	CTTCGGTACGCCTGGACA	[36]
recAR	ACACCGCCCCTCACACCA	[36]
salAF	TGAACTGCACCCTCATGCATCTCT	This study
salAR	CGATATGCTGCTTCACAATGGGCA	This study
syfAF	GTATGTGCTCGACGCTTATGA	This study
syfAR	GTCTTCGGCAGGTTCAGATAG	This study
sylAF	CCATCGGCAAGACCTGTAAT	This study
sylAR	TGGTTCTGACACCCAACTTC	This study
sypAF	ACGCGAAAGGACCAACTAC	This study
sypAR	CTCAAGGCGGTCTGCATATAA	This study

sypBF	GACCGTGCATGTGACCTATTA	This study
sypBR	CCACAGGTATCGAGCAGATAAA	This study
sypCF	TTCGGAAACCACCACCTATTC	This study
sypCR	AGCAGGTAGAACTGCGTATTG	This study
syrB1F	ATATCGTCTCTGCGCGTATTG	This study
syrB1R	GTGTAGATGATGTAGGCAGTGG	This study
syrB2F	AATGGTTGCCTGCAGTTCATTCCC	This study
syrB2R	TCCTTATCGATCTGCAACTGGCGA	This study
syrCF	GGTTATCGACCGCACATCTAAT	This study
syrCR	CACTGTCGCCCAGATTGTAA	This study
syrDF	GGTGTTCTCTGACTTCCATCTG	This study
syrDR	CCTCGATCTTGACCTTGTCTTC	This study
syrEF	CATGTCACCCTGGAACTGATAC	This study
syrER	GTGCGACAAACTCCCGATAA	This study
syrPF	CTATCGCTCAACGCCGTATC	This study
syrPR	TGCTCGCAGAAGAACCATT	This study
syrrFF	ATGAACTGCTCTTGACGCAGAGT	This study
syrFR	TGAGTACAAGTACCTGCAACGCGA	This study
syrGF	ATACGCCTGTCATCCACTTGACCA	This study
syrGR	AGCTGCACGTCAAGAACAGCTCTA	This study
syrRF	CACATGCAGGACCCGTTGTTGATT	This study
syrRR	AGGCTCTTGAGCAAGGACAACTCT	This study
